# Supramolecular interactions between catalytic species allow rational control over reaction kinetics†
†Electronic supplementary information (ESI) available. See DOI: 10.1039/c9sc02357g


**DOI:** 10.1039/c9sc02357g

**Published:** 2019-08-14

**Authors:** Abraham J. P. Teunissen, Tim F. E. Paffen, Ivo A. W. Filot, Menno D. Lanting, Roy J. C. van der Haas, Tom F. A. de Greef, E. W. Meijer

**Affiliations:** a Institute for Complex Molecular Systems , Eindhoven University of Technology , P.O. Box 513 , 5600 MB Eindhoven , The Netherlands . Email: e.w.meijer@tue.nl ; Email: t.a.f.d.greef@tue.nl; b Laboratory of Macromolecular and Organic Chemistry , Eindhoven University of Technology , P.O. Box 513 , 5600 MB Eindhoven , The Netherlands; c Schuit Institute for Catalysis , Eindhoven University of Technology , P.O. Box 513 , 5600 MB Eindhoven , The Netherlands; d Computational Biology , Eindhoven University of Technology , P.O. Box 513 , 5600 MB Eindhoven , The Netherlands

## Abstract

The adaptivity of biological reaction networks largely arises through non-covalent regulation of catalysts' activity. Such type of catalyst control is still nascent in synthetic chemical networks and thereby hampers their ability to display life-like behavior. Here, we report a bio-inspired system in which non-covalent interactions between two complementary phase-transfer catalysts are used to regulate reaction kinetics. While one catalyst gives bimolecular kinetics, the second displays autoinductive feedback, resulting in sigmoidal kinetics. When both catalysts are combined, the interactions between them allow rational control over the shape of the kinetic curves. Computational models are used to gain insight into the structure, interplay, and activity of each catalytic species, and the scope of the system is examined by optimizing the linearity of the kinetic curves. Combined, our findings highlight the effectiveness of regulating reaction kinetics using non-covalent catalyst interactions, but also emphasize the risk for unforeseen catalytic contributions in complex systems and the necessity to combine detailed experiments with kinetic modelling.

## Abstract 2

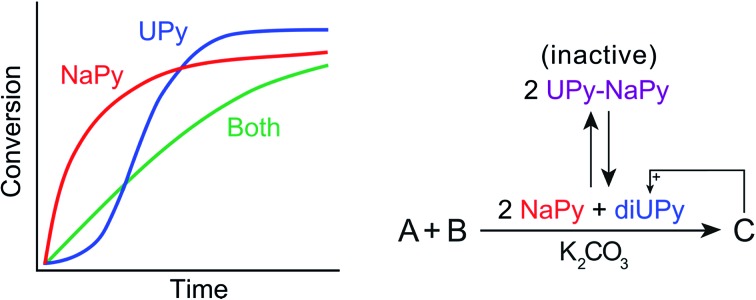
The non-covalent interactions between two phase-transfer catalysts allow tuning of reaction kinetics from bimolecular, to pseudo 0^th^ order, to sigmoidal. Kinetic models and DFT calculations are used to obtain detailed insight in the system.

## Introduction

The behavior of living cells is largely regulated through complex networks of biochemical reactions. Without regulatory mechanisms, the kinetics of such reactions would mainly be governed by substrate concentration, leaving cells poorly able to adapt to changes in their environment. By evolving enzymes whose activity can be controlled through the non-covalent binding of cofactors,^1^,^2^ Nature has created means to dynamically up or downregulate specific reaction pathways depending on the need for certain products. Such a strategy is especially effective when the enzyme and effector molecule are part of the same metabolic pathway, as the resulting feedback or feedforward loops often give rise to strong and non-linear responses.^3^ Besides using small molecules to regulate catalysis, Nature also employs interactions between separate catalysts to regulate reaction kinetics. An example is the organization of metabolically linked enzymes into dynamic clusters called metabolons.^4^ This strategy not only enhances local substrate concentration and helps to segregate metabolic pathways, but also allows for control over entire pathways through disruption of the metabolon,^5^ or a more nuanced competition between the enzymes therein.^6^–^8^ Combined, such dynamic interactions between catalysts and other reaction compounds – including other catalysts – give rise to unique regulatory control that is characteristic of living systems.

Regulatable catalysts have proven to be a useful element in synthetic systems as well. For example, the binding of metals^9^–^12^ and other atoms^13^,^14^ has been used to create catalysts which can be switched “on” or “off”, or whose enantioselectivity can be altered. However, the catalyst-ligand binding strengths in such systems are usually too large to allow for dynamic competition and the possibility to perturb the system by small changes in concentration, temperature, or solvent composition.^15^,^16^ In addition, this type of regulation simply alters the reaction's overall rate or selectivity, and not the shape of its kinetic curves, as is the case in natural systems (*e.g.*, the “sigmoidalness” of certain enzymes' reaction kinetics can be regulated by altering their sensitivity to autocatalytic feedback).^17^ To enhance synthetic catalysts' adaptability, single catalysts composed of multiple non-covalently bound molecules have been developed.^18^–^22^ Because such supramolecular catalysts are typically held together by relatively weak hydrogen bonds, their compositions – and thereby their activities and selectivities – are more open to gradual and dynamic regulation (*e.g.*, by inhibition through the addition of a competing binding motif).^23^–^26^ Furthermore, the dynamic nature of supramolecular catalysts enhances their susceptibility to interact with other reaction components, which facilitates feedback mechanisms and communication between otherwise distinct reactions. Overall, the advancement of life-like synthetic systems is expected to benefit from dynamic control over both the overall reaction rate as well as the type of kinetics displayed by a reaction, and this can be achieved by employing multiple catalysts that interact with each other as well as with different reaction components.

**Fig. 1:**
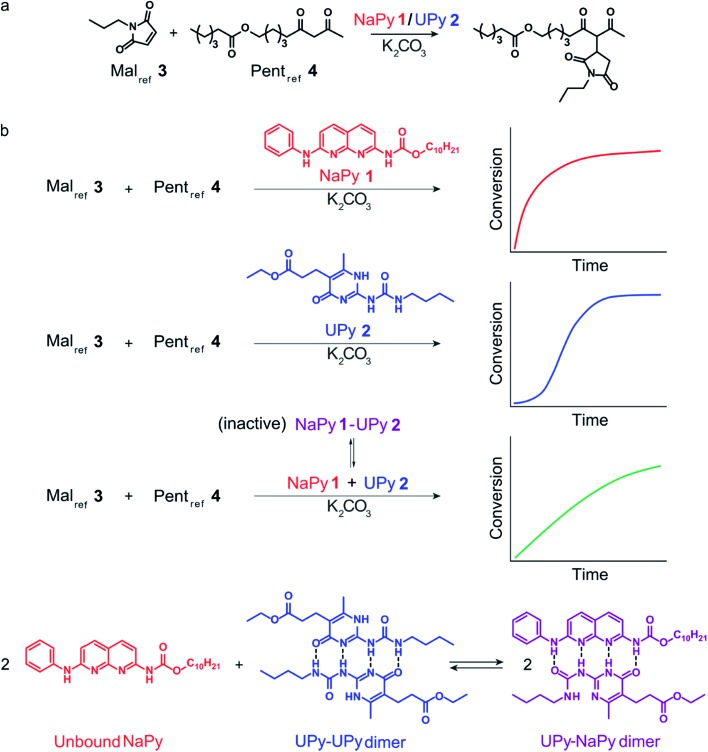
Chemical structure of the compounds used and their role in the Michael addition. (a) Structure of the substrates of the Michael addition (Mal_ref_**3** and Pent_ref_**4**), as well as the K_2_CO_3_ solubilizing phase-transfer catalysts used (NaPy **1** and UPy **2**). (b) The catalytic activity and type of kinetics associated with each species in the K_2_CO_3_ catalyzed Michael addition, and the equilibria between UPy **2** (*K*_dim_ = 6 × 10^7^ M^–1^ at 25 °C in CHCl_3_)^28^ and NaPy **1** (*K*_a_ = 5.2 × 10^5^ M^–1^ for UPy **2** and NaPy **1** in CDCl_3_, see Fig. S2† for experimental results of binding constant determination by ^1^H NMR).

Here, we present a combined theoretical and experimental study of a catalytic system in which the interactions between two complementary phase-transfer catalysts allow tuning of reaction kinetics ranging from bimolecular, to pseudo 0^th^ order, to sigmoidal. This system builds upon our earlier findings demonstrating that the supramolecular binding motif 1,8-naphthyridine (NaPy) is able to function as a K_2_CO_3_ solubilizing phase-transfer catalyst for the Michael addition, for example in the reaction between maleimide and 2,4-pentanedione in chloroform (Fig. 1a).^26^ It was shown that this reaction displays bimolecular reaction kinetics and that the overall reaction rate can be regulated by inhibition using the NaPy complementary ureidopyrimidinone (UPy) motif.^26^ We also showed that a fixed ratio of NaPy and ditopic UPy can be diluted while buffering the concentration of catalytically active free NaPy, thereby desensitizing the Michael additions' rate to dilution.^24^–^27^

We now show that UPy motifs functionalized with an ester moiety on their alkylidene position can also function as phase-transfer catalyst and that the interactions between such catalytically active UPys and the NaPy catalyst can be used to regulate the kinetics of the Michael addition (Fig. 1b). Interestingly, sigmoidal kinetics are observed when the Michael addition is catalyzed by ester functionalized UPys which results from the Michael product stabilizing the catalytically active complex between UPy and K_2_CO_3_ (diUPy·K_2_CO_3_), thereby enhancing the latter's rate of formation (autoinduction). Besides stabilizing the diUPy·K_2_CO_3_ catalyst, the Michael product can also act as an individual phase-transfer catalyst by forming a complex with K_2_CO_3_ (product·K_2_CO_3_), thereby giving rise to autocatalysis. Kinetic models and density functional theory (DFT) calculations are used to obtain insight in the catalytic species' structures and the interplay between them. It is shown that the non-covalent interactions between the UPy and NaPy catalysts can be used to regulate reaction kinetics from bimolecular to strongly sigmoidal (Fig. 1b). In addition, we examine the extent to which the kinetics can be controlled by optimizing the linearity of the kinetic curves, thereby creating pseudo 0^th^ order kinetics. The engineering of such bioinspired reaction networks containing interacting catalysts and multiple feedback loops will aid the development of autonomous chemical systems that sense their environment, processes chemical stimuli, and respond at the molecular level.

## Results and discussion

### Analyzing the bimolecular kinetics of the Michael addition catalyzed by NaPy and K_2_CO_3_

**Fig. 2:**
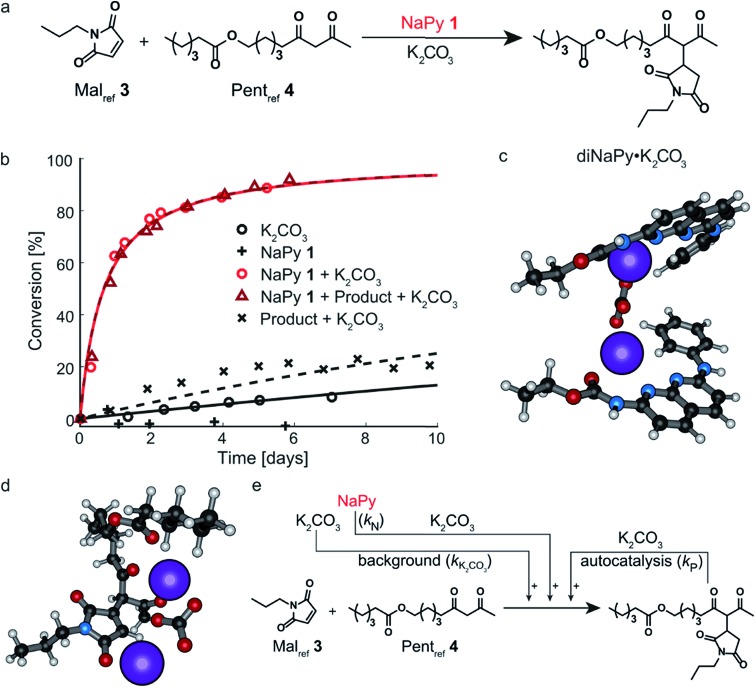
Experimental and computational data related to the Michael addition catalyzed by NaPy and K_2_CO_3_. (a) Schematic depiction of the NaPy **1** catalyzed Michael addition between Mal_ref_**3** and Pent_ref_**4**. (b) The conversion of the Michael addition between Mal_ref_**3** (*c* = 4 mM) and Pent_ref_**4** (*c* = 4 mM) in the presence of various combinations of K_2_CO_3_ (*c* = 36 mM), NaPy **1** (*c* = 8 mM) and additionally added product (*c* = 10 mM, symbols) and the best fits of the kinetic model based on bimolecular mass action kinetics (lines, lines with identical color belong to the same reaction, with the only difference that they are dashed when additional Michael product was added at the start of the reaction). The results show that the product does not significantly influence the rate of the NaPy catalyzed reaction. The reactions were performed in CDCl_3_ at room temperature, all components were combined simultaneously. (c) Optimized geometry as obtained from DFT calculations on two NaPys complexing K_2_CO_3_, showing how the nitrogen lone-pairs, aromatic rings and carbonyl oxygens of each NaPy coordinate to a potassium ion while the carbonate anion binds the two potassium ions together. (d) Optimized geometry as obtained from DFT calculations on the Michael product complexing K_2_CO_3_. (e) Schematic of the kinetic mass action model including the background reaction, autocatalysis, and NaPy catalysis. The formation of the product·K_2_CO_3_ and diNaPy·K_2_CO_3_ complexes was not included in the model as it is not required to obtain a proper fit of the data, instead their formation is viewed as being instantaneous.

In good agreement with our previous work on a structurally similar NaPy,^26^ both NaPy **1** and K_2_CO_3_ were found to be required for efficient catalysis between Mal_ref_**3** and Pent_ref_**4** (Fig. 2a and b). This NaPy and K_2_CO_3_ catalyzed reaction displays bimolecular kinetics and can be described by a mass-action kinetic model based on NaPy catalysis, background reaction catalysis by non-complexed K_2_CO_3_, and weak autocatalysis (*vide infra*). We propose that the observed catalytic activity of NaPy result from NaPy complexing K_2_CO_3_ (NaPy·K_2_CO_3_), thereby enhancing K_2_CO_3_'s solubility and catalytic efficiency.^26^ An optimized DFT structure of the proposed NaPy·K_2_CO_3_ complex (see ESI†) shows that the nitrogen lone-pairs of NaPy **1**, as well as its phenyl ring and carbonyl oxygen, can coordinate to a K^+^ ion, closely resembling naphthyridine complexes reported in literature.^29^–^31^ Although DFT calculations produced a stable structure assuming a NaPy : K_2_CO_3_ ratio of 1 : 1, a much higher stability was obtained for a structure comprising two NaPys per K_2_CO_3_ (diNaPy·K_2_CO_3_), likely because in that case each K^+^ ion is stabilized by a separate NaPy (Fig. 2c, see ESI† for the calculated stabilities of mono and diNaPy·K_2_CO_3_). Such a diNaPy·K_2_CO_3_ complex is also in agreement with our kinetic analysis, which shows that NaPy has a reaction order higher than unity (approximately 1.2, see Fig. S12F†). Combined, these data suggest that a mixture of mono and diNaPy·K_2_CO_3_ species is responsible for the catalysis observed, for clarity we will refer to this mixture simply as diNaPy·K_2_CO_3_.

During our investigation of NaPy's catalytic role, we found that reactions catalyzed by K_2_CO_3_ and pre-added product display slightly faster rates than those catalyzed by K_2_CO_3_ only (Fig. 2b). We therefore propose that in addition to NaPy, also the product is able to complex and solubilize K_2_CO_3_. A plausible structure of such a catalytic complex in which the Michael product binds K_2_CO_3_ (product·K_2_CO_3_) was provided by DFT calculations, showing coordination of several of the Michael product's carbonyl moieties to K_2_CO_3_ (Fig. 2d). Such product-mediated catalyst activation represents an uncommon form of ligand-acceleration,^32^ which has been classified both as autoinductive^33^ and autocatalytic.^34^,^35^ We chose to use the term autocatalysis to describe product·K_2_CO_3_-mediated rate acceleration, as this is in agreement with other systems in which the reaction product promotes its own formation by functioning as phase-transfer catalyst.^35^–^37^ Interestingly, no significant increase in reaction rate was observed when the diNaPy·K_2_CO_3_ catalyzed reaction was performed in the presence of additional Michael product (Fig. 2b). Subsequent kinetic analysis of these results showed that the high catalytic activity of NaPy **1** reduces the product's contribution to the overall conversion to just a few percent (see Fig. S13C† for computational fits and simulations). Combined, our results show that the kinetics of the NaPy catalyzed Michael addition are composed of diNaPy·K_2_CO_3_ catalysis, autocatalysis, and a K_2_CO_3_ background reaction, and that the overall kinetics of this reaction can be accurately described using a kinetic model that includes these contributions (Fig. 2e and S12E†).

### Analyzing the sigmoidal kinetics of the Michael addition catalyzed by UPy and K_2_CO_3_

**Fig. 3:**
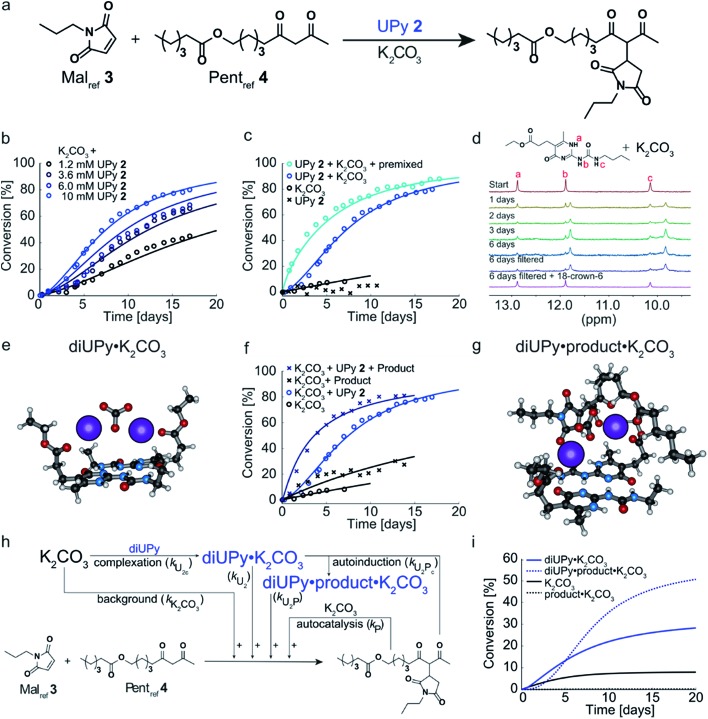
Experimental and computational data related to the Michael addition catalyzed by UPy **2** and K_2_CO_3_. (a) Schematic depiction of the UPy **2** catalyzed Michael addition between Mal_ref_**3** and Pent_ref_**4**. (b) Conversion of the Michael addition between Mal_ref_**3** (*c* = 4 mM) and Pent_ref_**4** (*c* = 4 mM) in the presence of K_2_CO_3_ (*c* = 36 mM) and various amounts of UPy **2** in CDCl_3_ at room temperature (symbols), and the best fits of the kinetic model based on mass action kinetics of UPy catalysis through diUPy·K_2_CO_3_ complex formation, autocatalysis by product·K_2_CO_3_ complex formation, and autoinduction as a result of the product binding and further activation of the catalytically active diUPy·K_2_CO_3_ complex (lines). All components were combined simultaneously. (c) Conversion of the Michael addition between Mal_ref_**3** (*c* = 4 mM) and Pent_ref_**4** (*c* = 4 mM) in the presence of K_2_CO_3_ (*c* = 36 mM), in the presence of UPy **2** (*c* = 10 mM), or when both are present (symbols) and the best fits based on the same kinetic model as in (b) (lines). Typically, all components were combined simultaneously, except for the “premixed” measurement where UPy **2** was stirred in a suspension of K_2_CO_3_ in CDCl_3_ for six days prior to adding the Michael substrates. (d) ^1^H NMR spectra of UPy **2** (*c* = 4 mM) in the presence of K_2_CO_3_ (*c* = 36 mM) in CDCl_3_, displaying the changes in the ^1^H NMR signals corresponding to the UPy NH protons over time and their recovery upon the addition of K^+^ complexing agent 18-crown-6 (*c* = 8 mM). (e) Optimized geometry of the UPy–UPy dimer complexing K_2_CO_3_ as obtained from DFT calculations. Note that the ester moieties of the UPys fold back over the dimer plane to coordinate to the K^+^ ions. (f) Conversion of the Michael addition between Mal_ref_**3** (*c* = 4 mM) and Pent_ref_**4** (*c* = 4 mM) in the presence of K_2_CO_3_ (*c* = 36 mM) and additional Michael product (*c* = 10 mM) and/or UPy **2** (10 mM) in CDCl_3_ at room temperature (symbols) and the best fits based on the same kinetic model as in (b) (lines). (g) Optimized geometry of the diUPy·product·K_2_CO_3_ complex as obtained from DFT calculations. (h) Schematic of the kinetic mass action model including the background reaction, autocatalysis, diUPy·K_2_CO_3_ complexation, and autoinduction. The formation of the product·K_2_CO_3_ complex was not included in the model as it is not required to obtain a proper fit of the data, instead its formation is viewed as being instantaneous. (i) Catalytic contributions of the background reaction, autocatalysis, UPy catalysis, and autoinduction in the Michael addition catalyzed by K_2_CO_3_ (*c* = 36 mM), Mal_ref_**3** (*c* = 4 mM), Pent_ref_**4** (*c* = 4 mM) and UPy **2** (*c* = 10 mM), simulated using the optimized parameters.

Having analyzed and modeled the K_2_CO_3_ background reaction, autocatalysis, and bimolecular NaPy catalysis, we turned our attention to the sigmoidal kinetics observed for the UPy-catalyzed Michael addition. While UPy homodimers are typically catalytically inactive, we discovered that UPys functionalized with an ester moiety on their alkylidene position (*e.g.*, UPy **2**) are able to solubilize K_2_CO_3_ and thereby act as phase-transfer catalyst for the Michael addition in CDCl_3_ (Fig. 3a and c). Surprisingly, the reactions catalyzed by UPy **2** and K_2_CO_3_ displayed sigmoidal kinetics, that is, an initial lag-phase followed by a strong increase in reaction rate (Fig. 3b). Interestingly, this lag-phase is only observed when all components are combined simultaneously *i.e.*, when UPy **2** and K_2_CO_3_ are combined several days prior to the addition of the Michael substrates the lag-phase is absent (Fig. 3c). This suggests that the lag-phase originates from the time required to form a catalytically active complex between UPy and K_2_CO_3_ (diUPy·K_2_CO_3_). In good agreement, mixing UPy **2** with K_2_CO_3_ generates new signals in the ^1^H NMR spectrum of UPy **2** which only stabilize after several days (Fig. 3d). When 18-crown-6 is added as a competing K^+^ complexing agent the typical UPy ^1^H NMR signals are immediately recovered, proving that the complexation of K_2_CO_3_ by UPy **2** is indeed a slow, non-covalent, and reversible interaction. Furthermore, the addition of a separate complexation step between UPy and K_2_CO_3_ in our kinetic model results in a similar lag-phase and a better fit of the data (see Fig. S14† for validation). UPys lacking the ester functionality do not show any changes in their ^1^H NMR spectrum upon mixing with K_2_CO_3_, nor any catalytic activity towards the Michael addition. The optimized DFT structure of the diUPy·K_2_CO_3_ complex shows a structure in which the esters of the UPys fold back over the plane of the UPy–UPy dimer and thereby coordinate to K_2_CO_3_, in good agreement with our experimental observations (Fig. 3e).

To investigate the influence of the Michael product on the UPy **2** catalyzed reaction, the reaction between Mal_ref_**3** and Pent_ref_**4** was performed in the presence of UPy **2**, K_2_CO_3_, and additional product, added at the start of the reaction (Fig. 3f). Compared to the reactions catalyzed by UPy **2** and K_2_CO_3_ only, this led to a much shorter lag-phase and significantly faster reaction rates. Although the Michael product can act as a separate phase-transfer catalyst (*vide supra*), our model shows that the autocatalysis – determined by analyzing the reaction catalyzed by Michael product and K_2_CO_3_ only, Fig. 2b – is not strong enough to explain the observed rate-acceleration. Instead, our kinetic model and DFT calculations suggest that the product stabilizes the diUPy·K_2_CO_3_ complex by forming a structure in which K_2_CO_3_ is chelated by both the UPy dimer as well as the Michael product (Fig. 3g). Although this diUPy·product·K_2_CO_3_ complex catalyzes the Michael addition with a similar rate constant as the diUPy·K_2_CO_3_ complex, it is more stable and formed significantly faster, thereby giving rise to rate acceleration. Such product-mediated catalyst activation is termed autoinduction (see Fig. S15† for validation of inclusion of autoinduction in the kinetic model, and Fig. S12E† and DFT results for the determined rate constants and complex stabilities). Combined, our results show that although the product can act as an orthogonal catalyst (autocatalysis), in this reaction it functions mainly as an activator for the diUPy·K_2_CO_3_ catalyst (autoinduction, Fig. 3h and i). This autoinductive mechanism is therefore the predominant cause of the sigmoidal kinetic curves observed for the UPy catalyzed Michael addition (see Fig. S14 and S15†).

### Tuning the Michael addition's reaction kinetics by combining NaPy **1** and UPy **2**

**Fig. 4:**
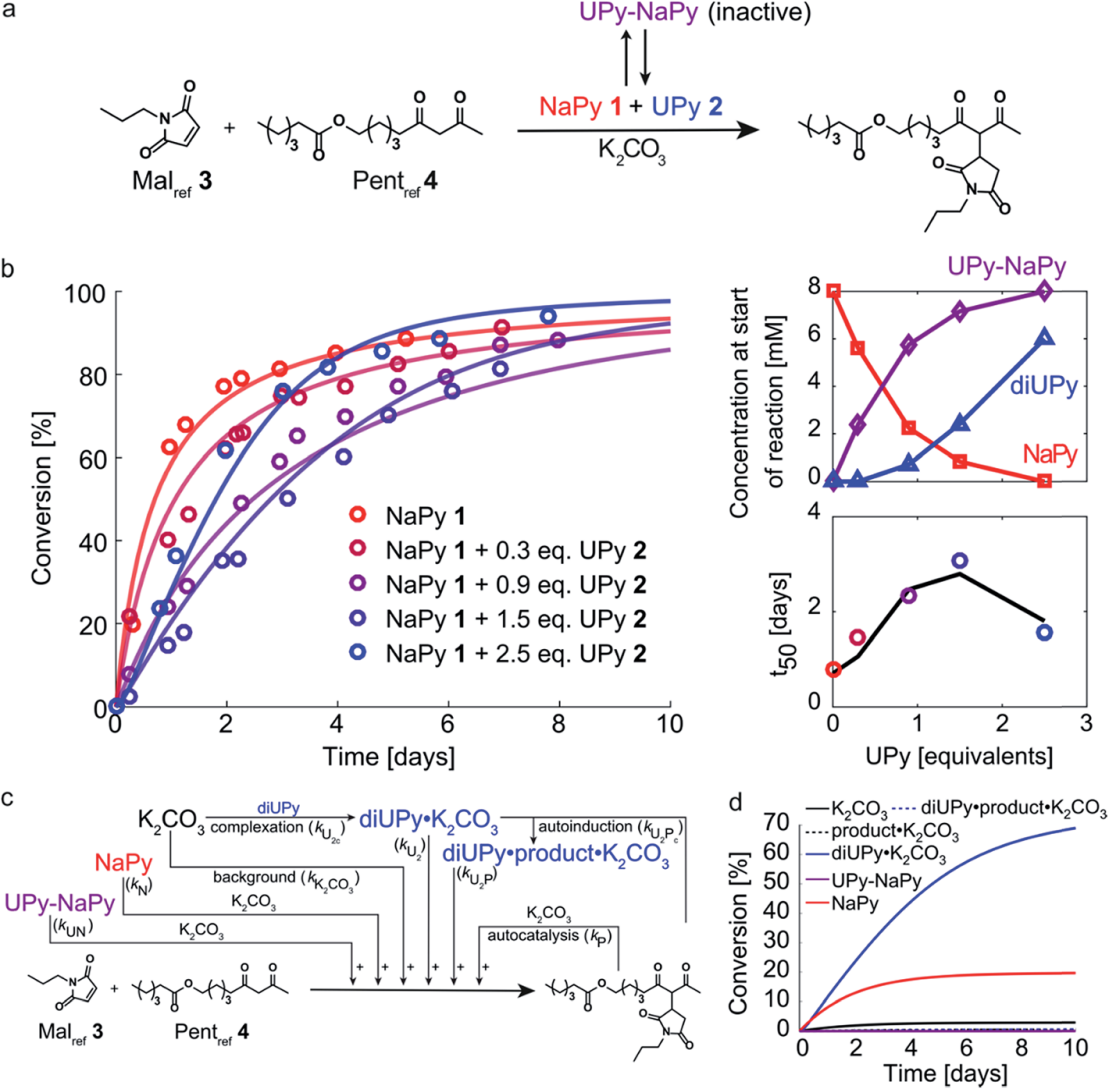
Experimental and computational data related to the Michael addition catalyzed by K_2_CO_3_ and combinations of NaPy **1** and UPy **2**. (a) Schematic depiction of the NaPy **1**, UPy **2** and K_2_CO_3_ catalyzed Michael addition between Mal_ref_**3** and Pent_ref_**4**. Part of the UPy and NaPy will form UPy–NaPy heterodimers in solution, which are catalytically inactive. (b) The conversion of the Michael addition between Mal_ref_**3** (*c* = 4 mM) and Pent_ref_**4** (*c* = 4 mM) in the presence of K_2_CO_3_ (*c* = 36 mM), NaPy **1** (*c* = 8 mM) and various amounts of UPy **2** in CDCl_3_ at room temperature (symbols). In addition, the best fits of the kinetic model based on mass action kinetics of NaPy, diUPy and UPy–NaPy phase-transfer catalysis, autocatalysis as a result of the Michael product functioning as an additional phase-transfer catalyst, and autoinduction caused by the Michael product binding and thereby activating the already catalytically active diUPy·K_2_CO_3_ complex (lines, see Fig. S12† for details on the kinetic model) are shown. The insets depict the speciation of UPy and NaPy at the start of each reaction and the time required to reach 50% conversion using the different equivalents of UPy **2**. All reactions were performed in CDCl_3_ at room temperature, all components were combined simultaneously. (c) Schematic of the expanded kinetic mass action model including the background reaction, autocatalysis, diUPy·K_2_CO_3_ complexation, autoinduction, NaPy catalysis, and UPy–NaPy catalysis. The formation of product·K_2_CO_3_, diNaPy·K_2_CO_3_ and UPy–NaPy·K_2_CO_3_ complexes was not included in the model as this is not required to obtain a proper fit of the data, instead their formation is viewed as instantaneous. (d) Catalytic contributions of the background reaction, autocatalysis, UPy catalysis, UPy autoinduction, NaPy catalysis, and UPy–NaPy catalysis in the Michael addition catalyzed by K_2_CO_3_, NaPy (*c* = 8 mM), and UPy (*c* = 12 mM = 1.5 eq.), simulated using the optimized parameters of the best fit.

Having analyzed the bimolecular kinetics resulting from NaPy **1** and the sigmoidal kinetics resulting from UPy **2** separately, we set out to examine the effect of combining both motifs (Fig. 4a). Previously, we have shown that binding of NaPy to any type of UPy – with or without ester functionality – inhibits diNaPy·K_2_CO_3_ catalysis through to the formation of catalytically inactive UPy–NaPy heterodimers.^24^,^26^ This ability of UPy to inhibit NaPy catalysis lead us to hypothesize that the interactions between catalytically active NaPy **1** and UPy **2** might be used to regulate the contribution of each catalytic species to the overall reaction rate, and thereby the kinetic profile of the Michael addition.

To test this hypothesis, the Michael addition between Mal_ref_**3** and Pent_ref_**4** was performed in the presence of NaPy **1**, K_2_CO_3_, and various amounts of UPy **2** (Fig. 4b). We observed that the addition of small amounts of UPy **2** (0.3 and 0.9 equivalents with respect to NaPy **1**), led to a decrease of the overall reaction rate compared to the reaction performed without UPy **2** present. Increasing the UPy **2** concentration to 1.5 equivalents did not lead to a further reduction in the overall reaction rate, but notably altered the shape of the kinetic curve from bimolecular to a more linear character. Interestingly, performing the reaction with even more UPy **2** present (2.5 equivalents) led to an increase in the overall reaction rate and slightly sigmoidal kinetics.

To obtain more insight into this system, a kinetic model was constructed containing background catalysis by non-complexed K_2_CO_3_, phase-transfer catalysis by binding of either UPy–NaPy heterodimers, UPy homodimers, or the Michael product to K_2_CO_3_, and lastly, autoinduction by the Michael product enhancing the diUPy·K_2_CO_3_ catalyst's stability and rate of formation (Fig. 4c). Interestingly, when we used the optimized parameters obtained from modelling the reactions catalyzed by K_2_CO_3_ and UPy only, we were unable to model those catalyzed by both K_2_CO_3_, UPy and NaPy. Therefore, all parameters used to model the reactions catalyzed by both UPy and NaPy were set free. This discrepancy seems to be caused by an activating role of NaPy on the UPy catalysis *vide infra*. Gratifying, the computational model revealed that an increase in UPy **2** concentration leads to a rise in the UPy homodimer and UPy–NaPy heterodimer concentrations, as well as a decrease in the free NaPy concentration (Fig. 4b). The effects of the increasing amounts of UPy **2** on the reaction kinetics can thus be qualitatively explained by a decreasing contribution of the bimolecular kinetics resulting from diNaPy·K_2_CO_3_ catalysis, and an increasing influence of the sigmoidal kinetics brought about by the diUPy·K_2_CO_3_ catalyst. Similarly, the changes in the overall reaction rate arise from the varying amounts of diNaPy·K_2_CO_3_ and diUPy·K_2_CO_3_ catalyst present.

Although the observed changes in kinetics can be qualitatively explained by the varying concentrations of diNaPy·K_2_CO_3_ and diUPy·K_2_CO_3_ catalyst, a detailed analysis of our results revealed a complex interplay between these species. First, our DFT calculations revealed that the catalytically inactive UPy–NaPy dimers are able to form a stable complex with K_2_CO_3_ (UPy·NaPy·K_2_CO_3_), and that this proceeds through a UPy-type mechanism, *i.e.*, the ester moiety on the UPy binds one of the K^+^ ions, while NaPy does not directly interact with K_2_CO_3_ (see ESI† for an optimized DFT structure of UPy·NaPy·K_2_CO_3_). This stability of UPy·NaPy·K_2_CO_3_ is somewhat surprising, as our kinetic analysis shows that it does not contribute to the overall catalysis (Fig. 4d). While we were not able to isolate UPy·NaPy·K_2_CO_3_ for further investigation, its lack of catalytic activity could be explained if multiple UPy–NaPy dimers are required to form an efficient phase-transfer catalyst. Such a structure comprising K_2_CO_3_ and several UPy–NaPy dimers might not be formed at concentrations high enough to produce a noticeable effect on the overall reaction rate, which agrees with the high reaction order suggested by our kinetic analysis (Fig. S12F†). Secondly, our kinetic analyses reveal that – although UPy and NaPy partially deactivate each other through the formation of UPy–NaPy dimers – the diUPy·K_2_CO_3_ catalyst itself has a higher catalytic activity and rate of formation when in the presence of NaPy **1** (Fig. S12E and F†). This could be explained by K_2_CO_3_ exchanging between fast forming diNaPy·K_2_CO_3_ and the more stable diUPy·K_2_CO_3_ (see ESI† for DFT calculated stabilities of all catalytic species). Lastly, our ^1^H NMR data suggests that binding of the Michael product to diUPy·K_2_CO_3_ (*i.e.*, the autoinduction) shifts the UPy–NaPy equilibria from catalytically inactive UPy–NaPy heterodimers towards catalytically active UPy homodimers and free NaPy (not shown, as quantification of this phenomenon was troubled by gradual deuteration of UPy and NaPy). Such a shift in equilibria would agree with the Michael products' stabilizing influence on the diUPy·K_2_CO_3_ catalyst as determined by DFT, and would represent an additional source of rate acceleration by generating additional free NaPy and diUPy phase-transfer catalyst. Combined, these results show that the non-covalent interactions between NaPy **1** and UPy **2** give rise to a complex catalytic system which cannot be fully explained by a simple linear combination of the NaPy and diUPy phase-transfer catalysts.

Our results show that increasing the ratio of UPy **2** to NaPy **1** allows regulation of the Michael addition's kinetics from bimolecular to sigmoidal, with moderately linear kinetics obtained at intermediate UPy **2** concentrations (*i.e.*, 1.5 eq.). However, the rate acceleration induced by UPy catalysis is not sufficient to counteract the influence of the decreasing substrate concentration on the reaction rate, and as a result all curves start to level off above ≈70% conversion (Fig. 4b). To examine the extent to which the kinetics in our system can be regulated – and test our kinetic model – we set out to optimize the linearity of the kinetic profiles. This specific goal was chosen because it requires delicate balancing of the biomolecular and sigmoidal contributions to the overall reaction rate, which will likely provide additional insight in the respective catalysts' properties. To achieve this, two goals need to be met. Firstly, it is essential that the reaction rates at higher conversions are increased, *i.e.*, the rate acceleration resulting from autoinduction needs to be enhanced. Secondly, the optimal amount of NaPy required to linearize the kinetics has to be determined.

### Enhancing the rate acceleration resulting from autoinduction

**Fig. 5:**
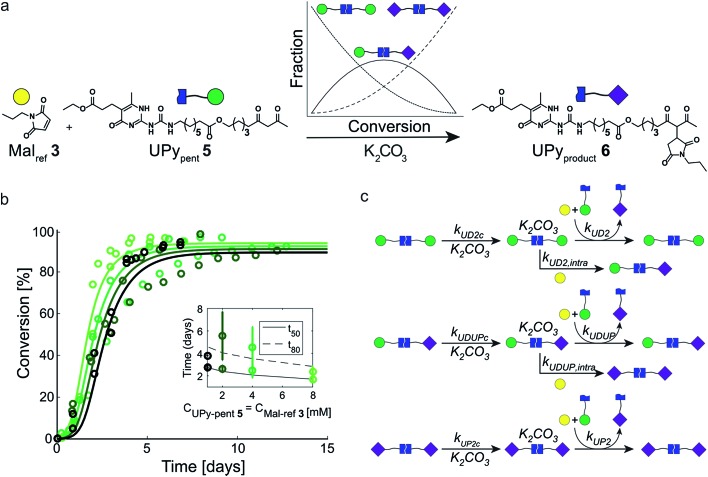
Experimental and computational data related to the Michael addition catalyzed by UPy_pent_**5** and K_2_CO_3_. (a) The reaction between Mal_ref_**3** and UPy_pent_**5**, showing how the fraction of each type of diUPy catalyst changes with conversion. (b) The conversion of the K_2_CO_3_ catalyzed Michael addition between equimolar mixtures of Mal_ref_**3** and UPy_pent_**5** (symbols) and the best fits of the kinetic model based on mass action kinetics of diUPy_pent_, UPy_pent_·UPy_product_, and diUPy_product_ K_2_CO_3_ complexation and subsequent inter- and intramolecular catalysis (lines). The concentration of K_2_CO_3_ is kept constant (*c* = 36 mM), while the concentrations of Mal_ref_**3** and UPy_pent_**5** are changed simultaneously (*c* = 1, 2, 4 and 8 mM). The reactions were performed in duplicate in CDCl_3_ at room temperature, all components were combined simultaneously. The results show that diluting both substrates by a factor eight does not notably reduce the reaction rate. (c) Schematic of the kinetic mass action model for the Michael addition between Mal_ref_**3** and UPy_pent_**5** including diUPy·K_2_CO_3_ complexation, and inter- and intramolecular catalysis, see ESI† for details on the computational model, fits of the experimental results, and obtained reaction constants.

In our search for ways to enhance the rate-acceleration induced by the autoinductive binding of the Michael product to diUPy·K_2_CO_3_ – and thereby the linearity of the kinetic curves – we noticed that reaction rates have been shown to increase when a catalyst is covalently linked to one of its substrates.^38^,^39^ We hypothesized that in our system this approach might not only increase the overall reaction rate, but also enhance the degree of rate-acceleration by bringing the Michael product in close proximity of the diUPy·K_2_CO_3_ catalyst. To test our hypothesis, we synthesized UPy_pent_**5** in which pentanedione is covalently attached to the UPy motif (Fig. 5a). Functionalizing UPy in this manner is expected to have several effects: (1) the linker increases the local concentration of substrate (pentanedione) around the diUPy·K_2_CO_3_ catalyst, which is expected to increase the overall reaction rate; (2) after the pentanedione has reacted, the local concentration of Michael product around the diUPy·K_2_CO_3_ is increased in a similar manner. Since binding of the product to the diUPy·K_2_CO_3_ catalyst is the driving force for the rate-acceleration in the UPy catalysis (*vide supra*), this is expected to lead to stronger rate-acceleration; and (3) during the reaction, UPy_pent_**5** gets converted to UPy_product_**6**, as a result, the UPy catalyst gradually changes from diUPy_pent_·K_2_CO_3_ to UPy_pent_·UPy_product_·K_2_CO_3_ to diUPy_product_·K_2_CO_3_ (Fig. 5a). Given their different structures, it is proposed that these catalysts have different activities as well.

To test the influence of covalently linking UPy to pentanedione on the reaction kinetics, UPy_pent_**5** was reacted with Mal_ref_**3**. Interestingly, the reaction between **5** and **3** is much faster compared to reactions between Michael substrates **3** and **4** catalyzed by similar amounts of UPy **2** (Fig. 3b and 5b). As the high activity of UPy_pent_**5** is proposed to result from intramolecular interactions, and the equilibrium between intra- and intermolecular contacts depends strongly on concentration,^39^,^40^ we investigated the influence of concentration on the Michael addition catalyzed by UPy_pent_**5** and K_2_CO_3_. Surprisingly, reducing the concentration of UPy_pent_**5** and Mal_ref_**3** by a factor eight resulted in only a slight decrease in reaction rate (Fig. 5b). This insensitivity could be described by a kinetic mass action model that includes the effects described (Fig. 5c), and revealed that the catalysts' efficiency increases from diUPy_pent_ (UD_2_) to UPy_pent_·UPy_product_ (UDUP) to diUPy_product_ (see Fig. S16† for calculated reaction constants).

### Optimizing the kinetic curves' linearity by tuning the NaPy concentration

**Fig. 6:**
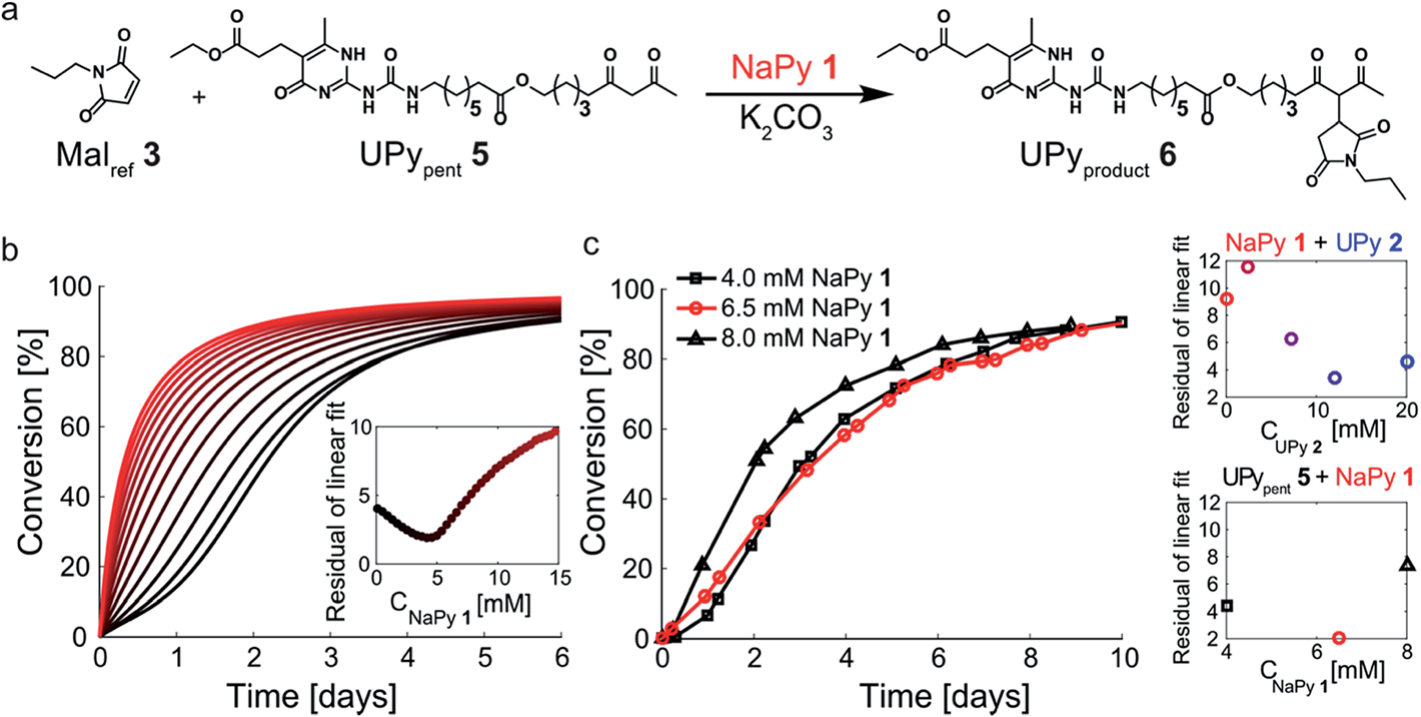
Optimizing the kinetic curves' linearity by catalyzing the Michael addition using K_2_CO_3_, UPy_pent_**5**, and various amounts of NaPy **1**. (a) Schematic depiction of the Michael addition between Mal_ref_**3** and UPy_pent_**5**. NaPy **1** and the various types of UPy dimers formed between UPy_pent_**5** and UPy_product_**6** all function as phase-transfer catalyst. (b) Simulated kinetic curves of the reaction between UPy_pent_**5** (*c* = 4 mM), K_2_CO_3_ (*c* = 36 mM) and varying amounts of NaPy **1**. The residuals of a linear fit up to 75% conversion were calculated, showing that the most linear kinetics can be obtained using a mixture of NaPy **1** (≈5 mM), Mal_ref_**3** (4 mM), UPy_pent_**5** (4 mM) and K_2_CO_3_ (36 mM). The residuals were normalized to the number of simulated points up to 75% conversion, to enable a concentration dependent comparison. (c) The conversion of the K_2_CO_3_ catalyzed Michael addition (*c* = 36 mM) between equimolar mixtures of Mal_ref_**3** (*c* = 4 mM), UPy_pent_**5** (*c* = 4 mM), and varying amounts of NaPy **1** (symbols). The reactions were performed in CDCl_3_ at room temperature, all components were combined simultaneously. The lines are to guide the eye. The inset shows the residuals of a linear fit of the data point up to 75% conversion for the reactions performed using UPy_pent_**5**, NaPy **1** and Mal_ref_**3**, as well as the reactions performed using UPy **2**, NaPy **1**, Mal_ref_**3** and Pent_ref_**4**.

Having successfully increased the rate-acceleration by linking pentanedione to the UPy motif, we investigated the influence of NaPy **1** on this system (Fig. 6a). The NaPy parameters obtained from the experiments using Mal_ref_**3** and Pent_ref_**4** were used to predict the kinetics of mixtures containing K_2_CO_3_, NaPy **1**, Mal_ref_**3**, and UPy_pent_**5**. These simulations indicate that changing the concentration of NaPy is an excellent way to alter the linearity of the kinetic curves (Fig. 6b). According to our predictions, the most linear kinetic curve can be obtained using a mixture of 4 mM UPy_pent_**5** and ≈5 mM NaPy **1**. Using these model results as a guide, we set out to experimentally determine the optimal ratio of NaPy **1** and UPy_pent_**5** for obtaining linear kinetics. While measurements performed using 4 mM UPy_pent_**5** and 4 mM NaPy **1** still displayed a lag-phase, increasing the NaPy **1** concentration to 6.5 mM resulted in near linear kinetics up to approximately 80% conversion (Fig. 6c). Further increasing the concentration of NaPy **1** to 8 mM lead to a decrease in the linearity of the kinetic curve and more bimolecular-like kinetics. These results show that, although absolute reaction rates cannot be predicted reliably, our model was successful in predicting the optimal conditions required to obtain pseudo 0^th^ order kinetics. Furthermore, the linearity of the kinetic curves was increased in comparison to the curves obtained using UPy **2** and NaPy **1** (Fig. 6c).

In our current study we have mainly altered the UPy–NaPy equilibria by changing the ratio of these motifs. However, it has been shown that many stimuli, such as light,^41^ pH,^42^ temperature,^28^ redox chemistry,^43^,^44^ and disulfide exchange^45^ can also be used to influence UPy–NaPy dimerization. In addition, changing the molecular structure of UPy or NaPy,^15^,^27^,^46^ or the addition of complementary binding motifs,^47^,^48^ have also proven excellent means of controlling these equilibria. Incorporating such mechanisms in our system will likely generate alternative means to enhance its applicability.

Nevertheless, our study also highlights the challenges associated with further increasing the system's complexity. It underscores the high likelihood of (unexpected) interactions arising in complex catalytic systems and the difficulties associated with fully comprehending and modeling these. For example, while our kinetic models could accurately describe the reaction progress curves, we were not able to precisely determine the value of all reaction rate constants. Furthermore, certain rate constants seem to vary with the complexity of the system (*e.g.*, the rate constants obtained from experiments with UPy only could not be used to satisfyingly predict the kinetics of reactions catalyzed by both UPy and NaPy). As explained, these limitations result from the exclusion of certain processes in our model, including transfer of K_2_CO_3_ between both catalysts and gradual shifts in the UPy–NaPy equilibria. Therefore, we believe that the further advancement of complex catalytic systems will increasingly rely on extensive kinetic modeling and the meticulous analysis of all interactions.

## Conclusions and perspectives

Non-covalently interacting catalysts are excellent tools to regulate reaction kinetics. It was revealed that the commonly used ureidopyrimidinone (UPy) binding motif is able to function as a phase-transfer catalyst in the K_2_CO_3_ catalyzed Michael addition and that such UPy catalyzed reactions display sigmoidal kinetics as a result of autoinductive feedback. The kinetics of this Michael addition can be regulated from bimolecular to strongly sigmoidal using the non-covalent interactions between UPy and the complementary NaPy motif, which was previously shown to also function as a phase-transfer catalyst. The duration of the lag-phase and strength of the rate acceleration can be controlled using a variety of parameters, such as the premixing time of UPy and K_2_CO_3_, the ratio between UPy and NaPy, and by covalently attaching UPy to one of the substrates. The results obtained were far from easy to predict nor explained without using theoretical models. Using these detailed kinetic models, as well as DFT calculations, it was possible to propose molecular structures for the catalysts while insight into their contribution to the overall reaction rate were obtained. These insights were subsequently used to examine the systems' scope – and kinetic model's accuracy – by optimizing the linearity of the kinetic curves, thereby mimicking the kinetics of a 0^th^ order reaction. Our findings suggest that catalytic supramolecular motifs are quite common and demonstrate how these can provide a direct link between covalent and non-covalent chemistry, a strategy that will benefit the advancement of life-like chemical systems. However, our results also emphasize the high likelihood of complex and hard to predict interactions arising in such catalytic systems and the accompanied necessity to computational model them.

## Experimental

Detailed descriptions about the synthesis and characterization of the new molecules are given in the ESI.† Also, the theoretical models are presented as well as the way the kinetics were measured. In short, the kinetic measurements on the Michael additions were performed on 5 mL CDCl_3_ scale in Wilmad screw-cap NMR tubes, diameter 10 mm, length 7 inch. Solutions were made by mixing premade 20 mM stock solutions of all organic compounds. The NMR tubes were shaken and rotated on a Hecht Assistant rotating mixer and removed for ≈20 minutes to measure their conversion by ^1^H NMR. Conversions were determined by measuring the decrease in signals associated with the maleimide and 2,4-pentanedione moieties. K_2_CO_3_ (99.995% purity) was ground and filtered (<0.125 mm) before use.

## Conflicts of interest

The authors declare no competing financial interest.

## Supplementary Material

Supplementary information

Supplementary information
