# Supplementary material for: Supramolecular interactions between catalytic species allow rational control over reaction kinetics
Source: Chem Sci. 2019 Aug 14;10(39):9115–24. doi: 10.1039/c9sc02357g (PMC6889839; doi:10.1039/c9sc02357g)
Supplement: Supplementary file 1 [file SC-010-C9SC02357G-s001.pdf]

Electronic Supplementary Material (ESI) for Chemical Science.
This journal is © The Royal Society of Chemistry 2019

Computational settings

All DFT calculations were performed using VASP. The PBE exchange-correlation functional was used in conjunction with the projector augmented wave approach. All structures were optimized to their local minima using the conjugate gradient algorithm as implemented in VASP. Optimization and other electronic settings are given below.

Settings

**Parameter**	**INCAR setting**	**value**
Cutoff energy	ENCUT	500
Precision	PREC	High
Smearing type	ISMEAR	0 (Gaussian smearing)
Smearing width	SIGMA	0.0005
Electronic convergence threshold	EDIFF	1E-5
Ionic relaxation threshold	EDIFFG	1E-4

K-point grid

For all calculations, only the Γ-point was used.

Geometries

Below, the geometries of the optimized structures are given. These geometries are represented as POSCAR/CONTCAR files. Further information with regard to the structure of these files is provided in the link below: http://cms.mpi.univie.ac.at/vasp/guide/node59.html.

Overview of obtained energies and molecules structures

System	Electronic Energy [eV]
UPy	-252.666
NaPy	-259.303
K_2CO_3	-33.965
Product	-369.544
diUPy	-507.276
UPy-NaPy	-513.399
NaPy + K_2CO_3	-293.652
diUPy + K_2CO_3	-541.583
UPy-NaPy + K_2CO_3	-547.663
diNaPy + K_2CO_3	-553.408
Product + K_2CO_3	-404.589
diNaPy + product + K_2CO_3	-923.720
diUPy + product + K_2CO_3	-912.445

	Reaction energy	Cumulative reaction energy
**Pathways**	**[kJ/mol]**	**[kJ/mol]**
UPy + UPy → diUPy	-187.183	
diUPy + K_2CO_3 → diUPy•K_2CO_3	-32.820	-220.003
diUPy•K_2CO_3 + product → diUPy•K_2CO_3-product	-126.877	-346.881
UPy + NaPy → UPy-NaPy	-137.695	
UPy-NaPy + K_2CO_3 → UPy-NaPy•K_2CO_3	-28.686	-166.381
NaPy + K_2CO_3 → NaPy•K_2CO_3	-36.972	
NaPy•K_2CO_3 + NaPy → diNapy•K_2CO_3	-43.591	-80.563
diNaPy•K_2CO_3 + product → diNaPy•K_2CO_3•product	-73.914	-154.476
Product + K_2CO_3 → product•K_2CO_3	-103.937	

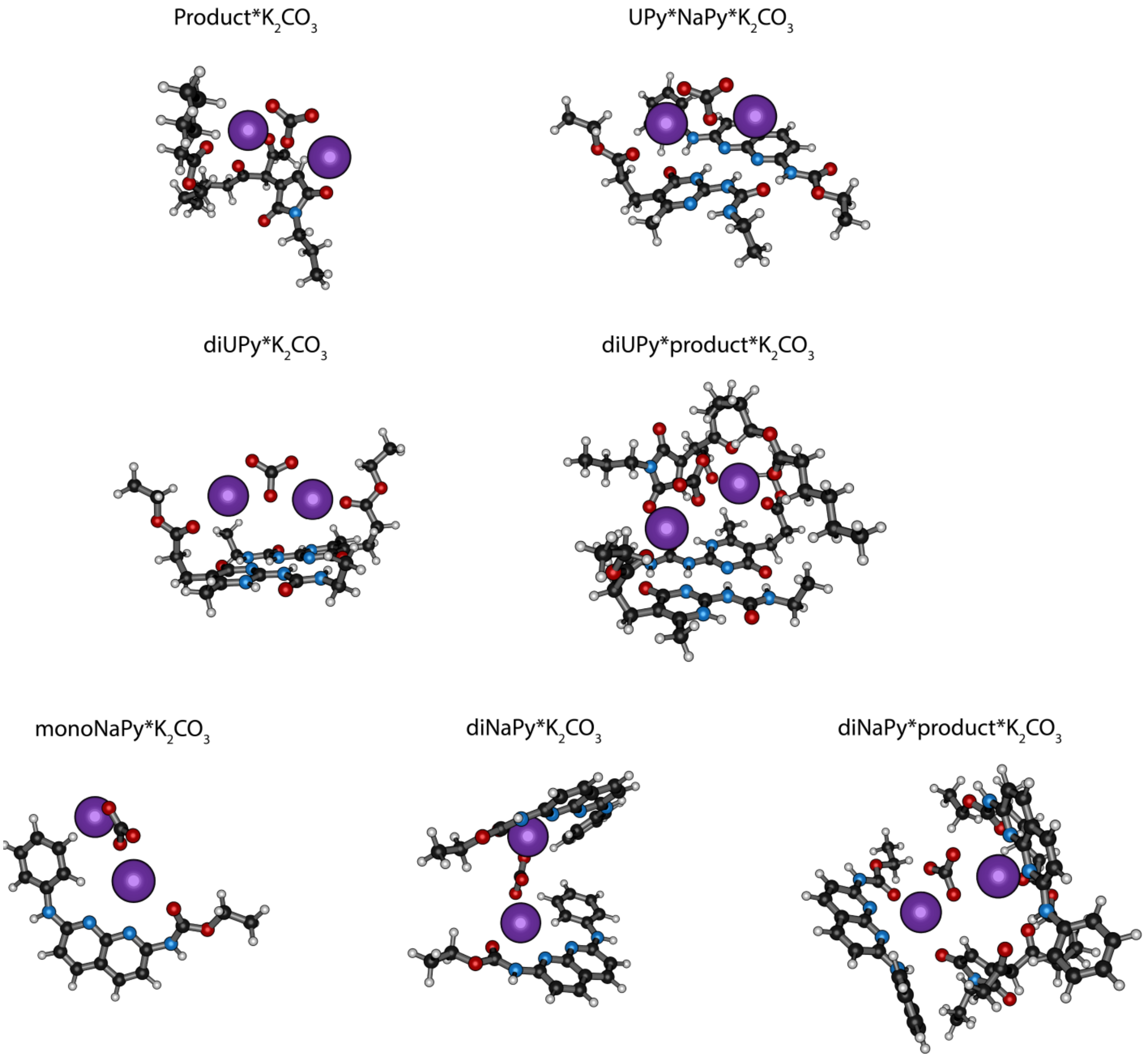

Figure 1: Optimized geometry as obtained from DFT calculations for the various phase-transfer catalysts.

```
UPy
   1.00000000000000
    25.8390500000000003    0.0000000000000000    0.0000000000000000
     0.0000000000000000   15.7686899999999994    0.0000000000000000
     0.0000000000000000    0.0000000000000000   15.8609100000000005
   C    H    N    O
    13    20     4     4
Direct
  0.5302223380125974  0.5897418510245399  0.5579642941534960
  0.4408275458047811  0.5761641247028775  0.5627532702150951
  0.4897962650304894  0.4485057265857404  0.5582407309246775
  0.5341006477660208  0.4958625518186784  0.5573708781209570
  0.4855766117304298  0.3536974245999975  0.5565730496035418
  0.5878528223919727  0.4593853750801225  0.5555507522165817
  0.6131801746856200  0.4676819541864686  0.4684014709720871
  0.6674228975582520  0.4316786781221301  0.4656848746765190
  0.7430297483244744  0.4178533223554134  0.3803084822017430
  0.7616772752985326  0.4442021508411448  0.2936816208503610
  0.3441696569756012  0.5738980516345269  0.5678555233317379
  0.2496500389623651  0.5943149595125561  0.5699566569584088
  0.2276659587159536  0.5844573637558982  0.4810348478172259
  0.4072494112033427  0.4596354678901906  0.5625602030717423
  0.4668107562373480  0.3300580404195692  0.6143131714762778
  0.4618212788779009  0.3329196295131170  0.5027754303030967
  0.5235253391330515  0.3234967835283503  0.5512594929775554
  0.6115565455114914  0.4951597383298638  0.6008750367582969
  0.5884734038060445  0.3928571868365358  0.5758140961598142
  0.5902130204084332  0.4350050318215674  0.4195995140067523
  0.6147187562688120  0.5347845516845192  0.4496367987704734
  0.7669557896694397  0.4448965223934056  0.4309881200565810
  0.7429618542835162  0.3485531845419170  0.3883931974280290
  0.7610515137280589  0.5134904444725751  0.2866703029067784
  0.8016555193127762  0.4223128442132841  0.2843428228986913
  0.7375134567777166  0.4164139073439800  0.2438499826030409
  0.2504587557229962  0.5326998067216351  0.6021667626497460
```

0.2255484118528384	0.6373594933067317	0.6078383982839370
0.2517467656841026	0.5411188432322281	0.4436716449722964
0.2256188138798527	0.6457119783123418	0.4480983689356092
0.3947449331260930	0.6786371978914404	0.5686241650639920
0.3079321903076931	0.6903045278717151	0.5643909662065895
0.1883083093546710	0.5580861418993759	0.4838204668862575
0.4808031048509798	0.6263072226993630	0.5616259951806137
0.4425103567263999	0.4901344722413303	0.5609540608409763
0.3919966574385758	0.6143802887009997	0.5659384608889620
0.3025094825519579	0.6270597634171241	0.5716258126615128
0.6903618467621815	0.4489780997771213	0.3902146717727340
0.6886182558933741	0.3919539891879395	0.5220729967793472
0.3397922511221780	0.4952271431084556	0.5672928341403541
0.5693612382510751	0.6350641644232101	0.5552297732775106

```
NaPy
   1.00000000000000
    27.5969799999999985    0.0000000000000000    0.0000000000000000
     0.0000000000000000   15.7130299999999998    0.0000000000000000
     0.0000000000000000    0.0000000000000000   12.9502600000000001
   C    H    N    O
    17    16     4     2
Direct
  0.5814817387558359  0.4986089334832076  0.5106956654379368
  0.4983447438126865  0.5166790955577238  0.5116698177136553
  0.5032056640048232  0.6071196706179176  0.5247353371806034
  0.5507239694348788  0.6406816230416632  0.5316311262478014
  0.5901042832296379  0.5875661587884784  0.5248035258820120
  0.4604202788388274  0.6561431234773837  0.5315581788128967
  0.4156728055186947  0.6176654276361274  0.5248606768282117
  0.4149796821492416  0.5278720484880255  0.5111038457249881
  0.3247375985469181  0.5095403039793651  0.5045550261765258
  0.2435360079180717  0.4594667856868077  0.4920274977609621
  0.2169075729822502  0.3759680379986019  0.4783175181003763
  0.6686620526097456  0.4514122327432464  0.4936243392290279
  0.6880463996692523  0.5172106832120946  0.4326578496474061
  0.7005501463745472  0.3925480565001853  0.5394015639130011
  0.7381088010008705  0.5248747318901672  0.4207776419948940
  0.7504512826272690  0.4001646142844504  0.5259038631142201
  0.7696673902130174  0.4668874620060277  0.4673751440890343
  0.3774014010985000  0.4179387314807795  0.4922246739115794
  0.5557440420306941  0.7089615628575976  0.5436059743508443
  0.6268206022610888  0.6118264292295817  0.5329557033340513
  0.4631490059766483  0.7249220796504781  0.5425666851622278
  0.2357435505307532  0.5045686988989525  0.4292037856158994
  0.2343209086352936  0.4908135878710997  0.5653982855890946
  0.1776396594155435  0.3877095017028307  0.4769297621579481
  0.2270873349732536  0.3449762594733461  0.4054252441682411
  0.2247608345599070  0.3319610807854664  0.5420816545150287
```

0.6639004194290461	0.5610573970394001	0.3926369151804298
0.6857044907588326	0.3411473170017760	0.5865435787441765
0.7524756972345130	0.5762634153281696	0.3730726280638262
0.7745154225388705	0.3539074748653216	0.5622288706668974
0.8087468693793114	0.4733950441301741	0.4577736930183660
0.6065471096831299	0.3790427031545893	0.5145715609693292
0.3818482962158865	0.6528376927916335	0.5297403785035418
0.5373543821305862	0.4645926951641537	0.5049672715436745
0.4541930770372510	0.4790714649870845	0.5051789847928536
0.3719867842287072	0.4814157229139271	0.5023902864893043
0.6187253587027883	0.4398154642415844	0.5072012334119514
0.3107041357939369	0.5828081344868001	0.5159203732905737
0.2950701996988913	0.4405785525537570	0.4916638386666209

```
Product
1.00000000000000
    21.0492470000000012    0.0000000000000000    0.0000000000000000
     0.0000000000000000   29.7692860000000010    0.0000000000000000
     0.0000000000000000    0.0000000000000000   15.2932980000000001
   C    H    N    O
    22    33     1     6
Direct
  0.3608158415052594  0.2712661087167163  0.4747092769222632
  0.4313613049681146  0.2728738156814072  0.4649590423697456
  0.4488415197124001  0.3148533134935348  0.4426709315612827
  0.3888886888256186  0.3431754474975320  0.4390980351493111
  0.2721896069119079  0.3290510145152973  0.4636836487490287
  0.2418009483846503  0.3384664232035741  0.3746007082597282
  0.1732245449984393  0.3545369010993046  0.3845992740678214
  0.5102146733766181  0.3374074723295760  0.4225470663874170
  0.5281836762711323  0.3715672536221275  0.4974476836892618
  0.5688626851906533  0.3077397725384002  0.4053770758206313
  0.5861613776630198  0.2716218732655596  0.4703109863067345
  0.5357811300597204  0.4192229242201361  0.4661099001131040
  0.5566271540749772  0.4539130685005152  0.5337430491462557
  0.5446275457454296  0.5015313270475026  0.4994231492575493
  0.5730010727049530  0.5392108361903958  0.5553786453366630
  0.5514238631651486  0.5846380106073583  0.5211598482767620
  0.5740709563137578  0.6623555026267419  0.5362169438144589
  0.6204594114628685  0.6964238840401366  0.5725695245266007
  0.5927302484550192  0.7438045571063866  0.5787925246220103
  0.6432956203459689  0.7799504779778615  0.5933370668727984
  0.6156808843534773  0.8273429422034962  0.6016117925805097
  0.6666382444236399  0.8637325245641675  0.6092129118070330
  0.4600164873229949  0.2430146261161356  0.4751112277400169
  0.2701656030501737  0.3593929820228105  0.5047578408616673
  0.2470021481406731  0.3018589660465640  0.4978117503437679
  0.2435740866202274  0.3076959938359551  0.3347069424310219
  0.2701532991515608  0.3640672276230338  0.3403613437020802
```

0.1517550959244183	0.3618718350363095	0.3207227219435055
0.1435392781220401	0.3290686411005270	0.4169505369300206
0.1708036636995632	0.3854326230828486	0.4239327605688305
0.5034659652204554	0.3575802204276837	0.3629837471296965
0.6377923471058119	0.2667882163601826	0.4696441123538930
0.5691761892586963	0.2794892613223313	0.5363941053663430
0.5643584309296541	0.2397391853778828	0.4494741891018176
0.4890788831687499	0.4281234538413635	0.4377896728013347
0.5683013789837531	0.4184237127453442	0.4093472971194745
0.6073114630306037	0.4492676722000538	0.5493555274812494
0.5306271187158099	0.4484848407323022	0.5953056528595397
0.4928398120656837	0.5065098970094920	0.4930080029126535
0.5639137573424662	0.5044700771052456	0.4325343621419554
0.6252704689502522	0.5374223687087043	0.5535380363166055
0.5587975153149858	0.5353498520545740	0.6243035363198347
0.5009697365060437	0.5909960141378262	0.5353691545744512
0.5574515117002545	0.5869937725964559	0.4497499396101641
0.6390093428734898	0.6844483520231472	0.6356202058407446
0.6614757820311115	0.6963521521196807	0.5274298291793760
0.5662941767269968	0.7509469762987105	0.5182696699162547
0.5574635419813569	0.7448897942334339	0.6320834956721842
0.6713732955994355	0.7721409648770947	0.6524324800515047
0.6772633581568722	0.7792062470487688	0.5382636412526731
0.5852670873634541	0.8342636293696948	0.5444338618197239
0.5840318133196930	0.8286880802576463	0.6590466341102943
0.6455348993851618	0.8973235673948373	0.6161331627313975
0.6973881498018437	0.8579925375869479	0.6663246789675129
0.6973206766799365	0.8642259106796403	0.5509443201850440
0.3383443641959133	0.3149838675175386	0.4579383058992812
0.3855665594661773	0.3836303491311734	0.4239491016705704
0.3274693581099146	0.2390480665895067	0.4936133683399223
0.5348311216429057	0.3589268481781878	0.5727257906291535
0.6012274109569052	0.3155799915017894	0.3406128390002872
0.5894860008459548	0.6197548627314619	0.5614679694563058
0.5294088216252316	0.6708679119293874	0.4880010990308735

```
K2CO3
   1.00000000000000
    10.1745500000000000    0.0000000000000000    0.0000000000000000
     0.0000000000000000   15.1000800000000002    0.0000000000000000
     0.0000000000000000    0.0000000000000000   12.4926499999999994
   C    O    K
     1     3     2
Direct
  0.5016166668634465  0.5002664241402416  0.4601981373352465
  0.5016894395319085  0.4994405477601391  0.5688877938606735
  0.4991685870727470  0.5758799294495438  0.4120560474378590
  0.5037822108678575  0.4253561842001508  0.4104025529180418
  0.5008529215319999  0.6618193869186236  0.5758620556015537
  0.4929001741320436  0.3372375275313080  0.5725934128466259
```

```
diUPy
   1.00000000000000
    28.5438399999999994    0.0000000000000000    0.0000000000000000
     0.0000000000000000   22.0218699999999998    0.0000000000000000
     0.0000000000000000    0.0000000000000000   16.2097900000000017
   C    H    N    O
    26    40     8     8
Direct
  0.4166448818404240  0.5888755013496439  0.5565683074836131
  0.4980313864081116  0.5950964568709626  0.5621128395153150
  0.4545209931229603  0.6876668843078976  0.5641670081571365
  0.4135882113415008  0.6553052412436196  0.5599886343764159
  0.4595315268066463  0.7553210704311637  0.5687696535887348
  0.3654712296831705  0.6830985863807968  0.5592411833883726
  0.3433831601618014  0.6833709600664142  0.4729184909479263
  0.2953091945416522  0.7123929985983342  0.4718583251413093
  0.2265939057304762  0.7273818376837037  0.3904440562303523
  0.2079428473474657  0.7091324749835202  0.3066514456677301
  0.5842057696935236  0.5994161828843451  0.5669733638864946
  0.6692888951315731  0.5882146288224573  0.5702600526119561
  0.6926914484603915  0.5925580849430088  0.4855684709723929
  0.5826135736800140  0.4071464357188819  0.5526718639059763
  0.5012399970416692  0.4006582959397920  0.5569734635634980
  0.5448114155862042  0.3081635651548435  0.5519405452871319
  0.5857320481092541  0.3406836945655850  0.5511993434076063
  0.5399017374678613  0.2404195732525479  0.5501156328622544
  0.6339992721150121  0.3133203343357577  0.5491528553608567
  0.6570127194200835  0.3189997177376521  0.4639937746169082
  0.7063023732907209  0.2938105686973662  0.4618627558569223
  0.7749255821729425  0.2847719200665190  0.3785639395336812
  0.7914460657924143  0.3037327057463212  0.2936869945109983
  0.4150651166733412  0.3963335733667076  0.5614505770147667
  0.3299993653331160  0.4076335603078464  0.5633872446662755
  0.3089965908259620  0.4022618033167842  0.4768608549825102
  0.5297852249675812  0.6764516043042598  0.5681419454635664
```

0.4768140994046612	0.7684683326413594	0.6267911275136485
0.4816781704760016	0.7719934594186876	0.5180388583858248
0.4256726626734764	0.7783956457675232	0.5654518726823564
0.3428096674395332	0.6562248993891568	0.6002133659834209
0.3658043903138842	0.7294873816816478	0.5839055031872693
0.3655014861539297	0.7086059652578378	0.4288600152918027
0.3406387295112329	0.6369066202585130	0.4485972106759353
0.2045999885875280	0.7103303455748067	0.4411236050664538
0.2292331336032463	0.7769581353520105	0.3974415958383316
0.2060619648481364	0.6595123455109659	0.3007463786094883
0.1724863785214164	0.7275917311145557	0.2983394288523777
0.2302152820840424	0.7267305762587766	0.2568477537946904
0.6671083725593383	0.6332914051011904	0.5989383990856909
0.6899782803340840	0.5587320072076094	0.6112201778818070
0.6731037371997948	0.6233552720051478	0.4449075803750910
0.6945490785314101	0.5477692583092066	0.4558699194779571
0.5409877597212132	0.5204320179393728	0.5602146774582671
0.6183628093190892	0.5162418366608325	0.5604993254334907
0.7285824590338597	0.6101180867331882	0.4918341699734348
0.4695265087505771	0.3191489320221593	0.5566937738325197
0.5227979941410095	0.2240917974180818	0.6067927245552560
0.5177096054873727	0.2263938060232140	0.4979817020342172
0.5738196737136304	0.2177575744278639	0.5442901541875826
0.6560195881435824	0.3376722439264716	0.5939480750775210
0.6336796959714306	0.2655517435165156	0.5684040829928918
0.6364238638405442	0.2950877472493868	0.4164630188590708
0.6582174421554284	0.3668834633166033	0.4448340543665040
0.7965194056286967	0.3044389002857267	0.4280260214234145
0.7751221380274547	0.2351706744190234	0.3866700960267797
0.7904135114821002	0.3533257164659918	0.2867675006947248
0.8277271260918713	0.2885801076440495	0.2842026160662258
0.7695485853048215	0.2835032832295646	0.2452267967257728
0.3312340773004215	0.3629066191664239	0.5932698797855142
0.3083090309887008	0.4377190763663625	0.6018205623715974
0.3294916673346311	0.3705955107961736	0.4389239029805976

0.3084112282874734	0.4466238769190992	0.4458476463515196
0.4583644815342472	0.4753555377686270	0.5598411984623253
0.3810196884474859	0.4797105240758727	0.5590252545060398
0.2728245308179827	0.3852684605709152	0.4803222384461456
0.4599975001407166	0.5608094150804347	0.5585962796991237
0.4964009277459422	0.6566618705171452	0.5648745710242695
0.5412474858865791	0.5679764080633172	0.5628704576390724
0.6220640419055267	0.5633003736174299	0.5665278803097895
0.5392628841344539	0.4350536195656815	0.5560779684226489
0.5028882756594055	0.3390625583008853	0.5548525620996517
0.4580705619885778	0.4277662160489259	0.5599340747150674
0.3772752027605140	0.4325502495055399	0.5631677086294112
0.2732163321455815	0.7012437708819249	0.3989820428460548
0.2781074747636895	0.7423827524463281	0.5275835464358664
0.5858668970411968	0.6563918442905943	0.5711888754275526
0.3800446049530611	0.5571033294060175	0.5522023015735544
0.7271051626871328	0.3066037224502018	0.3882975647794523
0.7255082433501306	0.2656061183632689	0.5172836015290642
0.4132939268257990	0.3392853690030361	0.5617868896032983
0.6192176515004981	0.4390632035920460	0.5510477869518455

```
UPy-NaPy-complex
   1.00000000000000
    28.4000699999999995    0.0000000000000000    0.0000000000000000
     0.0000000000000000   22.1164999999999985    0.0000000000000000
     0.0000000000000000    0.0000000000000000   16.0055200000000006
   C    H    N    O
    30    36     8     6
Direct
  0.5542509230248575  0.6278153833545995  0.5428688530820676
  0.4724452462607681  0.6393760515168804  0.5435393498084637
  0.4769572985783725  0.7033068973288070  0.5559673936979052
  0.5227798206319277  0.7279319174361394  0.5625467708536285
  0.5613040631208203  0.6912106577485355  0.5556160039204233
  0.4355673343871223  0.7380632925300222  0.5622399363556986
  0.3922336359624581  0.7110142478117281  0.5556000626503392
  0.3906931843145960  0.6474938436302283  0.5415703255672459
  0.3036135451582360  0.6420625652055897  0.5382416739892770
  0.2226011410740952  0.6165662657171583  0.5298717317094059
  0.1922172334126397  0.5606172942411872  0.5222231749694054
  0.6396542715712136  0.6035630947489701  0.5298758807952174
  0.6559911312832830  0.6498394254405183  0.4770068106407156
  0.6727240697665874  0.5675016473176721  0.5724200706811693
  0.7041981818785078  0.6607536455260300  0.4691976449710995
  0.7208064197830150  0.5783886243888847  0.5634132071559286
  0.7369024277612690  0.6253770070319756  0.5124234783888747
  0.5457358738453029  0.4392987771315010  0.5422129904358899
  0.4629711957209275  0.4415616534606674  0.5098939978758383
  0.5018007952916659  0.3494320416072290  0.5048893654682551
  0.5444653032207929  0.3749547630984775  0.5268864523232411
  0.4951821118265286  0.2832626757238885  0.4868279872098887
  0.5909048568101767  0.3423209580071086  0.5325123204343861
  0.6206422238205330  0.3536721029108615  0.4539715247231941
  0.6686560720019795  0.3241567722547098  0.4565071582170297
  0.7416870802771250  0.3157032852673224  0.3834643275501076
  0.7639768731793566  0.3391784247209717  0.3039484099304247
```

0.3777251040870280	0.4601076709633615	0.4789472631710407
0.3232840263306662	0.3789071834837317	0.4411046679969834
0.2941236138074992	0.3612590852390000	0.5176840821812158
0.3496924458128806	0.5719664911804274	0.5142010440826531
0.5268767211339226	0.7763224348685357	0.5742065882534076
0.5968213836260785	0.7088665535415444	0.5624697723048034
0.4383391601489736	0.7867978062239739	0.5723273179986863
0.2158975750059484	0.6490610337079374	0.4791522862777406
0.2169300192663476	0.6403229134237776	0.5893947740077705
0.1549139944250538	0.5735182908531903	0.5261649731928534
0.1980317539773084	0.5376755028641624	0.4622690445619042
0.1997441247875079	0.5284824500526238	0.5727989658727909
0.6309967932573666	0.6763540515222314	0.4405299592323763
0.6601458546493545	0.5308485125599950	0.6121627739839007
0.7162539334740337	0.6967972979596859	0.4278347690655545
0.7458805149437610	0.5499911712621808	0.5970120731551809
0.7745245613642479	0.6340186636211705	0.5059044421514532
0.5847033845060600	0.5434250368298207	0.5490550630016233
0.3594665706905821	0.7360603556419983	0.5601073675034031
0.4736952234521018	0.2623841764652039	0.5362855202332634
0.4756801373375218	0.2777518022255409	0.4280147173428180
0.5284060725192431	0.2584220156308743	0.4823720265703449
0.6103845555367462	0.3592008516049005	0.5870951213364148
0.5861171651942013	0.2936013917897932	0.5420161095751745
0.6023730472542913	0.3370374357291215	0.3976889941538729
0.6256330438886940	0.4025029747315722	0.4442284535995491
0.7616187909296220	0.3289504907612466	0.4394878280352723
0.7386049950919035	0.2661234191852715	0.3838400582897633
0.7668337393912411	0.3886909638465501	0.3048920361903681
0.7995585204072840	0.3202591593983101	0.2972916146164148
0.7433214778306545	0.3259908430106727	0.2488750843519159
0.3050525098165927	0.4144192181725715	0.4056803955922394
0.3278819404955389	0.3398092763294439	0.3993903513466439
0.2892815631074963	0.4004190173302231	0.5589823093617990
0.3112786608175785	0.3250247604938818	0.5534962983660395

0.4270184682259027	0.5236652925326186	0.5131140897835061
0.3987629672484415	0.3732611651177751	0.4694581877762488
0.2591465738842000	0.3451482557644810	0.4983761097874768
0.5046387645711305	0.5182652884691108	0.5364135635752002
0.5109603475831598	0.6027661432262645	0.5381159449218469
0.4293870190408388	0.6125649984772917	0.5362406508902000
0.3482239656489545	0.6173850632691025	0.5319563833946406
0.5916816887448906	0.5891515581607353	0.5385269511749688
0.5035296717510389	0.4705613893909971	0.5314099762598494
0.4611466455744362	0.3825232773736164	0.4976592946650143
0.4237787655274560	0.4774341129843616	0.5014103838795465
0.3700465484957996	0.4012662311050810	0.4620729971388903
0.2930531252671565	0.6947448815434106	0.5532375977180193
0.2713965023545217	0.5972858975723069	0.5253973774296852
0.6946638376358870	0.3415423997402826	0.3891765280440319
0.6828385305035799	0.2892925248528041	0.5100902278148223
0.3465791930644677	0.4997758305455008	0.4753124286419336
0.5821020925486747	0.4676020722120570	0.5633602567417718

```
NaPy-K2CO3-complex
   1.00000000000000
    26.3891099999999987    0.0000000000000000    0.0000000000000000
     0.0000000000000000   21.4276000000000018    0.0000000000000000
     0.0000000000000000    0.0000000000000000   12.0038900000000002
   C    H    N    O    K
    18    16     4     5     2
Direct
  0.5952384039500223  0.5814712490833359  0.5318848807235581
  0.5079831852091904  0.5905097187969771  0.5239966716130631
  0.5108746858688343  0.6570512662872964  0.5370340205414420
  0.5593108255225874  0.6844355448688543  0.5516198259235517
  0.6013679092264220  0.6472942395387511  0.5490722259733798
  0.4657392203463085  0.6921173043411960  0.5330683511588998
  0.4203352532825756  0.6619653173816091  0.5168216100316777
  0.4209101849584350  0.5959417666671381  0.5063350617733385
  0.3555910528884483  0.5091579563872372  0.5060250996987287
  0.2788075315372938  0.4501955269666126  0.5057440923420260
  0.2224673178182871  0.4625253219659174  0.4965067935409635
  0.6535568207416532  0.4859979337818394  0.5116249733999986
  0.6198440911007691  0.4357135738381261  0.5220612675842986
  0.7052243352214639  0.4738850913603165  0.4902021755686193
  0.6377824463462420  0.3745018359159719  0.5126762166534032
  0.7227460764083105  0.4129100166532313  0.4805809994402322
  0.6891017994629640  0.3627459381851379  0.4921777004596159
  0.5220072348758155  0.3032486354907115  0.4321518302532161
  0.3448809739881897  0.6000283662784065  0.4722047711217855
  0.5624333275826429  0.7347848233834936  0.5636551417203355
  0.6392286563492201  0.6669691349759569  0.5594420372087400
  0.4672992957464028  0.7427513874635401  0.5426745488564910
  0.2895824136120445  0.4331002450937603  0.5887304007697234
  0.2920396731721746  0.4160105734981913  0.4439254362207982
  0.2016097075438922  0.4190203630727968  0.5120571806698968
  0.2101634633793929  0.4972666007496473  0.5581414803365821
  0.2123021523152161  0.4794764947337488  0.4130966481867049
```

0.5800667066477311	0.4449979889447049	0.5373855356920966
0.7316316031927999	0.5129170796186884	0.4813932921633224
0.6111374561001278	0.3356524693202991	0.5204879755508519
0.7628909306724594	0.4046825026743799	0.4644692069747569
0.7028775085730212	0.3148389707493351	0.4866666698194841
0.6709045814458986	0.5775214809069062	0.5254682536176317
0.3846657986268201	0.6875802202653178	0.5135527815357757
0.5501536941380021	0.5536252585645827	0.5225045055327951
0.4624448801700402	0.5609628936009881	0.5100742641028561
0.3729993818167527	0.5690272498090958	0.4883373234429246
0.6402713769221455	0.5489739313244519	0.5239494670521735
0.3798736118675199	0.4635388586048781	0.5357111998245030
0.3046192617154595	0.5092218985418525	0.4857987416963083
0.4894622736744723	0.3023210207668575	0.5115135486776909
0.5236044392820901	0.3528684244737507	0.3640262299953342
0.5548659521343690	0.2588320722284201	0.4162311318700945
0.4734567779118368	0.4200608996703373	0.5005561833892630
0.5956657266536329	0.3112805531753610	0.2583722472910489

```
UPy-NaPy-k2co3-complex
   1.00000000000000
    27.5969699999999989    0.0000000000000000    0.0000000000000000
     0.0000000000000000   21.6859199999999994    0.0000000000000000
     0.0000000000000000    0.0000000000000000   20.3805399999999999
   C    H    N    O    K
    31    36     8     9     2
Direct
  0.5635003229591645  0.6240726621596392  0.5573819978740805
  0.4794598035752519  0.6362622019771192  0.5566663989331878
  0.4841394168507788  0.7011944364528374  0.5688120413908941
  0.5313289532177625  0.7259173916337858  0.5754879831411520
  0.5709011461454232  0.6883377700400178  0.5695691040942124
  0.4415843622218853  0.7365027216986356  0.5749592879503587
  0.3968800380373825  0.7091059809165168  0.5690820257335190
  0.3952616805503157  0.6447876940004603  0.5557579033173548
  0.3054840666443867  0.6382201944935488  0.5558161039468715
  0.2221885646322776  0.6108627389133755  0.5508347456512300
  0.1915417170074173  0.5534876179880220  0.5424956501942786
  0.6514148110605554  0.5996783360931035  0.5474178571627614
  0.6679538864932472  0.6470551062789862  0.5059171264863129
  0.6856514751972500  0.5634939885395674  0.5811951223543577
  0.7174802178714131  0.6586986201230665  0.4998431019644152
  0.7350765895345297  0.5751009097205800  0.5741369301921402
  0.7513549927195906  0.6229900406683037  0.5338723333500488
  0.5541268655351240  0.4304160955196824  0.5538159333648627
  0.4697318895942948  0.4352829182409071  0.5241781831023368
  0.5081000929657928  0.3401805356578448  0.5222129350558824
  0.5520420801673372  0.3648342851527542  0.5418898580279335
  0.4996764427813940  0.2729619468494157  0.5078131102008382
  0.5973578036012985  0.3275443979848655  0.5540107030412403
  0.6441961450776759  0.3481470815652031  0.5188163631538941
  0.6409896592215952  0.3557901542621848  0.4454613783967825
  0.6859530075917650  0.3565912568553398  0.3456242418327353
  0.7338941408945313  0.3336355262184955  0.3192931940256823
```

0.3824584862550068	0.4559657969037794	0.4992125208594383
0.3246881718821623	0.3728603621111521	0.4756494758771457
0.2963905339613148	0.3551651965177725	0.5374514444437295
0.4931123949641276	0.5453855062511359	0.2788184490421210
0.3531916615017807	0.5683142809765197	0.5319975696873027
0.5356385077984133	0.7749345196224448	0.5864395013276853
0.6074686611484923	0.7059273416626295	0.5764337470465768
0.4444617439875911	0.7859037913906516	0.5847465867537398
0.2146964841350142	0.6454193763224453	0.5126134243564060
0.2163721122857280	0.6330808628892085	0.5986867504479133
0.1529839035706471	0.5657719653451029	0.5457497110013684
0.1979928130103946	0.5318234220616488	0.4945876537462461
0.1994988463053849	0.5195313650687577	0.5809875866697616
0.6420531942994101	0.6739003018447132	0.4773323234809915
0.6728939412258045	0.5264685444443735	0.6130299439301107
0.7296695851142195	0.6955851719379590	0.4673812510961732
0.7610744436450181	0.5467020208196062	0.6008925414220803
0.7900241923042300	0.6321463398339082	0.5286871436921748
0.5948449186237202	0.5379672532123136	0.5617616620222907
0.3631684990822202	0.7345170755942406	0.5741140130359049
0.4806170895292812	0.2509495806051543	0.5491275407766267
0.4760562534216070	0.2686348645751151	0.4647017921226530
0.5329905286518557	0.2470056021047899	0.4986800230518331
0.6055939106028201	0.3292150249570460	0.6069458510767592
0.5903712180339785	0.2788872928781682	0.5426751221146363
0.6546246660482451	0.3939206508327860	0.5377452016223246
0.6738809466911501	0.3162323815947977	0.5305732809266226
0.6548978354970448	0.3353526304868051	0.3205883693325698
0.6825513765617565	0.4069625073114103	0.3407616956641901
0.7356534279758972	0.2830560750589007	0.3194929365349573
0.7382875365857497	0.3495771385764540	0.2684377764981730
0.7642687530118791	0.3517166707113161	0.3482821206940402
0.3052594738153987	0.4088747694203737	0.4483895922399977
0.3286223450674224	0.3327394048045099	0.4430409716838442
0.2913354192915170	0.3955189573444016	0.5693119617248010

0.3154666982876498	0.3193714406530638	0.5656149816492372
0.4338309035447519	0.5196857734599086	0.5263677849152149
0.4024968690310933	0.3667687614605921	0.4961045692047807
0.2604354843969603	0.3371714164091094	0.5242276362255621
0.5132820801383375	0.5122619548356313	0.5466676258954348
0.5190126448992829	0.5988700735967972	0.5518597678200647
0.4350678966690000	0.6093808010092870	0.5494260963763443
0.3515064135323417	0.6140334662637267	0.5478110010826144
0.6020561086402365	0.5845265243222201	0.5537867466328221
0.5117513184028740	0.4636602867288350	0.5419545467774078
0.4671412635351733	0.3751198788494643	0.5145432279062031
0.4299697090357213	0.4726483231335641	0.5170706760810361
0.3731589508353401	0.3957088962094038	0.4899787684291229
0.2944091365487251	0.6914448790753178	0.5696164279521508
0.2725963789083912	0.5921859988187357	0.5457931827575717
0.6833521670393758	0.3415961243689767	0.4154330641607693
0.6055905648504704	0.3752940145606133	0.4153714498283982
0.3515859495373541	0.4977805771687818	0.4930244643919644
0.5912734550779299	0.4586016114349851	0.5731136802050797
0.4859680631984827	0.5321860965995334	0.3431144402500548
0.4629840003584312	0.5823847999865206	0.2503860847783611
0.5294200303916243	0.5205461143275696	0.2491186827392512
0.5582480108447057	0.4642062773174451	0.3464654894664870
0.4131418542600331	0.5974392533332962	0.3482704545314978

```
diNaPy-k2co3-complex
   1.00000000000000
    27.1168499999999995    0.0000000000000000    0.0000000000000000
     0.0000000000000000   21.6137599999999992    0.0000000000000000
     0.0000000000000000    0.0000000000000000   20.7432899999999982
   C    H    N    O    K
    35    32     8     7     2
Direct
  0.5844563822556641  0.6916418799146880  0.4290191139946913
  0.4993622556618078  0.6953616253332748  0.4208553426942822
  0.5020100824484240  0.7204026098905822  0.3567863106817592
  0.5493908369865610  0.7311792601858823  0.3300438835267993
  0.5903075391935493  0.7171357802644704  0.3655162462152715
  0.4577433877223808  0.7327182404257787  0.3236477294048712
  0.4132946414605940  0.7206900184189635  0.3531999825661492
  0.4141217414729431  0.6961358933471514  0.4166391087376644
  0.3521351812513321  0.6668178005998969  0.5037267135616194
  0.2782649724755765  0.6439924192103278  0.5620854856760382
  0.2231076916573957  0.6446741559555564  0.5506732344675445
  0.6415771655297250  0.6518192998956965  0.5190842652473967
  0.6084452841105271  0.6350524809016953  0.5678127903856262
  0.6924388351094262  0.6417555036321266  0.5284631077394378
  0.6257024614419996  0.6081759525555638  0.6247583213493302
  0.7094825287023717  0.6154957691315521  0.5855238582814991
  0.6761669004935501  0.5985822815120928  0.6339326963757012
  0.5160147114767879  0.5132242944748711  0.6395889480673094
  0.6640235982380471  0.3487872040838536  0.5292056032729997
  0.6315418768681794  0.3871226030684902  0.5633560420387707
  0.7138624372268476  0.3458311742607374  0.5481402124950563
  0.6482582076299767  0.4207545937791168  0.6165571367035481
  0.7304811867545253  0.3800447386192360  0.6006841257105716
  0.6976733425651328  0.4172826888649627  0.6354239505501859
  0.6067885727057706  0.2947599949857926  0.4483833709710842
  0.5215900439160456  0.2905231353682613  0.4435045666798837
  0.5231039987523093  0.2512922072415970  0.3876327802875714
```

0.5699802713969904	0.2346547149339316	0.3627403365704279
0.6115792932912084	0.2561045873351174	0.3924662129659996
0.4782076748732153	0.2316381490607240	0.3602139815999897
0.4343472710709972	0.2505736104656666	0.3872266668946110
0.4363805770273127	0.2899198554514769	0.4419635378489740
0.3767661846394623	0.3396150458141141	0.5209565655583326
0.3058955544820091	0.3861088126929673	0.5699175016105135
0.2503819825397829	0.3827014466259276	0.5628110917653704
0.3388367568897666	0.6917785613970333	0.4121982960432781
0.5526732671366305	0.7503052375756331	0.2815048510842792
0.6272678209659017	0.7247668312676765	0.3462281675288493
0.4590616608681546	0.7516248952589295	0.2748726181109007
0.2894166106598614	0.6762878221158841	0.6003540473506208
0.2918398503440695	0.5977268980611540	0.5752942251099674
0.2038016401608774	0.6306680885759985	0.5948993495608098
0.2103325759860465	0.6911887662062629	0.5374029808828590
0.2127106958611412	0.6124611130247780	0.5120285360860459
0.5693354404742199	0.6424639281715324	0.5606117300035049
0.7184907425186661	0.6541500454647413	0.4903113336558711
0.5980730336018697	0.5935521321915286	0.6601828518302717
0.7489604207480655	0.6075743198452904	0.5916980772767834
0.6894540100090925	0.5772527199158224	0.6783118464661270
0.6578870639115071	0.6883580639346314	0.4319978624640657
0.3783329161876521	0.7297502060354795	0.3289287292180450
0.5936170561789098	0.3911938913381466	0.5473885512031437
0.7394601992489769	0.3163386989884128	0.5213603704238344
0.6212669510842996	0.4492447280362729	0.6423564652926723
0.7692556601012438	0.3772647822327602	0.6145602025919521
0.7106642997446492	0.4437391870973548	0.6768574099277305
0.5723390421368697	0.2051226091217053	0.3201410393773444
0.6481868797443930	0.2443081855615695	0.3743405232561043
0.4786012327007067	0.2016348458147011	0.3178423099337909
0.6804447991749817	0.2944387984715344	0.4525679341050850
0.3989091673545402	0.2360015925772592	0.3674841983395804
0.3613646794441139	0.2994978204057975	0.4357970970547695

0.3195787494660401	0.4339931761588217	0.5673488467158665
0.3188280902361574	0.3659909925076832	0.6155189237151657
0.2327268359991486	0.4100180009531859	0.6010648781741882
0.2384159140424029	0.4013020485253271	0.5160679476843876
0.2372747635319283	0.3347696954276547	0.5668168778515297
0.5403341704934692	0.6814826055241279	0.4560749932483009
0.4548905436195156	0.6837313962053091	0.4496707777015112
0.3672773074314442	0.6845455925575410	0.4430834741864394
0.6281030656275289	0.6783905387059369	0.4597401637565755
0.6508098040508827	0.3132149586813357	0.4751461025708917
0.4777732654841396	0.3093339876960669	0.4695774944746087
0.3902689822855400	0.3095011673758731	0.4650623380444333
0.5632051126374169	0.3115609143078646	0.4730898238263669
0.3773361339448403	0.6565080410845279	0.5513643087594279
0.3016688417651095	0.6626559511273162	0.5021199605927382
0.5415572753461673	0.4666353122652737	0.6604290443530736
0.4853805639253630	0.5048603422194182	0.5904953169607238
0.5199561741318878	0.5678152364899507	0.6648288704600361
0.4026791211296564	0.3529709459644121	0.5670308654655213
0.3272320309429153	0.3516191331586588	0.5165695784967661
0.4975267244115283	0.3877573186102454	0.5862239975040511
0.4716873828313046	0.6200980474633770	0.5726529887822485

```
di-NaPy-k2co3-product-complex
   1.00000000000000
    26.1067000000000000    0.0000000000000000    0.0000000000000000
     0.0000000000000000   25.8878799999999991    0.0000000000000000
     0.0000000000000000    0.0000000000000000   26.8407200000000010
   C    H    N    O    K
    57    65     9    13     2
Direct
  0.3979822153326385  0.5131778774651667  0.4093195401286865
  0.4542733405835725  0.5129501408581257  0.4089210889948137
  0.4704679037893203  0.5542324332825282  0.3811687973337203
  0.4249298600604058  0.5838084666512509  0.3645116285187677
  0.3294746506954707  0.5738013604220576  0.3769201261535677
  0.3058950026284240  0.5595344953788028  0.3264886429001630
  0.2501892222185190  0.5773514433019727  0.3228147318166604
  0.5227955634786969  0.5731827915264882  0.3674709998087113
  0.5448004992431262  0.6100389273532937  0.4071795730945219
  0.5595291031205968  0.5283469943505712  0.3559608719895871
  0.5480581516357628  0.4985168827431223  0.3089371212637856
  0.5634981604111630  0.6621788746622946  0.3882634919837171
  0.5662823345840081  0.7042606084412345  0.4281630833381098
  0.5135945239273234  0.7291992586210092  0.4392217418437905
  0.5025680976866778  0.7374721847592712  0.4948655316376978
  0.4920081862043113  0.6860830028185820  0.5200597373246405
  0.4877057240913820  0.6519172507226748  0.6022746404022009
  0.4760962654534716  0.6648528059987838  0.6560299148604837
  0.4718196382125521  0.6176177666035382  0.6898202288699018
  0.4589125572147310  0.6324453884073996  0.7435619911266663
  0.4544598120568449  0.5860857753705934  0.7786883118717368
  0.4397982689058967  0.6012783613136572  0.8318676554451382
  0.4848693455905184  0.4283856516756820  0.5658631499277448
  0.4251619220882530  0.3168472333947831  0.3900612691644341
  0.3853877543689833  0.2919724322841291  0.4632169256373491
  0.3988408324513280  0.2388832327457289  0.4555594948927104
  0.4264295967516477  0.2265861737492930  0.4116822526335686
```

0.4397998143953846	0.2648962620792693	0.3787667656744235
0.3835214488168798	0.2028106635312527	0.4921668063756461
0.3568903404681641	0.2193994155068291	0.5333949338044718
0.3457544709252040	0.2731905801922659	0.5376494615432303
0.3031631484552743	0.3378071903514182	0.5940591617757679
0.2662541940639754	0.3825949229444933	0.6626969260443322
0.2438711341198091	0.3704586589760294	0.7133783918807710
0.4687951399424674	0.3591171350361383	0.3173257901281978
0.5168677397121678	0.3346186199602821	0.3181053642837514
0.4545899280612348	0.3878328606049539	0.2750597451098916
0.5492526796184199	0.3378792624570105	0.2768678269825524
0.4875166488852933	0.3913773958244216	0.2342808856111653
0.5348511751470235	0.3661089875538640	0.2347269287628240
0.7135311226922059	0.5335915219344697	0.4795397528786023
0.6944536510351349	0.5008135943781152	0.5576240052818437
0.7470505200632303	0.4876072616351597	0.5665182377723030
0.7830851603312016	0.5003322924503258	0.5290646372752668
0.7670801320459101	0.5235781640003342	0.4858480083188139
0.7591096690603840	0.4616827655404812	0.6113370548169873
0.7208466934526995	0.4506179086950081	0.6448542162751781
0.6698892988216021	0.4666410253502254	0.6332891628566251
0.5830145092453820	0.4750183881466395	0.6720677901745381
0.5007957331530966	0.4535423586269264	0.7050592623300417
0.4808202132301017	0.4288110695821098	0.7523290891585307
0.7223548824554592	0.5742332691431337	0.3956977214214914
0.7616860180302140	0.6103450308910908	0.4042406735645470
0.7094752694321884	0.5615492057633530	0.3462777523172380
0.7879614407744826	0.6323249803902203	0.3642558551389161
0.7355491715219743	0.5843004384067250	0.3065739387427943
0.7751409777506171	0.6195510096798100	0.3152001742672274
0.3283739333177347	0.6160028971381546	0.3819881596774393
0.3076563191870213	0.5556883410143872	0.4073283644281772
0.3082587157589557	0.5173618214886033	0.3215303798893118
0.3289053079616401	0.5773166817691242	0.2967036457456906
0.2335023024427009	0.5675615460749759	0.2864409255305880

0.2263678896654114	0.5591042325980760	0.3517122120994012
0.2470766631712822	0.6194691793807297	0.3275480419712128
0.5178174578409330	0.5951340366690243	0.3324837830250244
0.5641228984731205	0.4594823638209380	0.3112246122123628
0.5069209990882526	0.4968996242658787	0.3008833382399629
0.5667099958150554	0.5188161924364948	0.2776819325387985
0.5394946716548851	0.6742805870964280	0.3565905787251504
0.6018648924852315	0.6542581902130701	0.3723372045684059
0.5935794163218781	0.7341756195742647	0.4162641762956755
0.5823576027886468	0.6869837018332964	0.4622020500166741
0.4828213297017608	0.7050063739570592	0.4233969709035719
0.5107757197114884	0.7664319140081592	0.4196442127990855
0.4693259675151897	0.7632036456067072	0.4995521412752487
0.5353652847587357	0.7565582514835143	0.5130520273416344
0.5237592124006342	0.6587262800685663	0.5155009898337218
0.4575159641334696	0.6676920489963162	0.5043596887341606
0.5065886419726718	0.6910778058213525	0.6691623213799112
0.4407485315229171	0.6881302322888599	0.6567644829496870
0.4421201682739235	0.5915384758770249	0.6750095830783286
0.5078000860045612	0.5954919708249182	0.6883318787116687
0.4884169639034491	0.6591385073981469	0.7580196086663271
0.4225619707133761	0.6543330029913011	0.7442488236110215
0.4258557884026156	0.5588318085977971	0.7636874763403552
0.4911525616858702	0.5649711716455978	0.7790100565726662
0.4371475881192810	0.5673651559798258	0.8563930403740235
0.4682341001870536	0.6277790595496752	0.8481124107807794
0.4024707363876344	0.6210490714279542	0.8327175452312233
0.3138148658557539	0.2613956584995454	0.6068050443823700
0.4366014129580461	0.1864178434530145	0.4040429908570702
0.4600385497019128	0.2560442204214485	0.3443750705751034
0.3930308677069070	0.1620021350719275	0.4875291327890083
0.2380185767113753	0.4003260868553720	0.6375600220836248
0.2994790256886554	0.4086061911452257	0.6650877132625744
0.2312982895572809	0.4064363773868832	0.7313307315356118
0.2105919872083669	0.3446923434800960	0.7102245543477936

0.2724274668585900	0.3520089793689508	0.7375962909999758
0.5292652459089148	0.3141648791330434	0.3514895463431065
0.4173006220608738	0.4069099162509104	0.2743418448843512
0.5864928492730007	0.3187820432307152	0.2781258078591731
0.4758293150369755	0.4136947740387016	0.2017214775718155
0.5604029595623781	0.3686267991859049	0.2026805254671167
0.4159303879576149	0.3902238510010228	0.3673419972785897
0.3446824872763540	0.1924197665855029	0.5622693293880169
0.6388485925921927	0.4234372515213183	0.6901417627582748
0.8234307649868902	0.4905210980908381	0.5344052612306857
0.7939045626517798	0.5321823769442506	0.4559537938033130
0.7987442477299729	0.4506473133907010	0.6189388587254269
0.4841994440007697	0.4365110493139452	0.6710764880534009
0.4944438381004853	0.4954778801606636	0.7045504495959607
0.4390974429160981	0.4341832288094978	0.7543210188670203
0.4886592438986402	0.3871151565714670	0.7528107470225487
0.4979742296444292	0.4463073090296303	0.7857401573623704
0.7708974235214222	0.6217636794116099	0.4423057223273895
0.6792696627622571	0.5332594909018167	0.3397130731870502
0.8183208669666494	0.6602901262529041	0.3715904496238522
0.7252234233843119	0.5738067485333004	0.2684878838469193
0.7956862767267322	0.6369759594564378	0.2840736404429202
0.6566320608362077	0.5443552774428060	0.4288049357430484
0.7288820216374641	0.4308621512188118	0.6798850060489493
0.4764924084953857	0.4842419248739402	0.4292557014682012
0.3822698807063367	0.5572401926102293	0.3827643730738147
0.3986845880419473	0.3296321907043234	0.4307504373075721
0.3590117053657891	0.3079889381265120	0.5040901228949940
0.3195967569109155	0.2888654210338838	0.5804316348458721
0.4352791901859430	0.3573657446599561	0.3582863096225656
0.6786634339204273	0.5231647266302638	0.5145530602442727
0.6570897337147610	0.4907539578267938	0.5915773460868282
0.6320798160304449	0.4544202573654100	0.6679429496693620
0.6944989893115093	0.5521739939251953	0.4350854957094223
0.4236608192018383	0.6243650140571537	0.3399429706744442

0.3676608812985143	0.4813831048171862	0.4278326562429384
0.5482987965452841	0.5967328136239811	0.4508167942376071
0.5968507546929519	0.5181905090496247	0.3818982655974177
0.4830956793898090	0.6945336233384558	0.5729287965485070
0.4997429288607008	0.6093365266709224	0.5864088171169396
0.4813985801179798	0.4521136332859403	0.5227084355732327
0.4442466388147916	0.4096222352932771	0.5866662842691803
0.5290363527553986	0.4252170835488693	0.5870974035099016
0.3048140119633793	0.3774480038299375	0.5695886227139696
0.2832275048434254	0.3340009957917084	0.6410340815815352
0.5684160702409432	0.5156069912126668	0.6537117846623685
0.5558936659531677	0.4440188107225342	0.7029952059436081
0.5559809629907424	0.5190305088427355	0.5500729525435718
0.3843402981081405	0.4176349895696141	0.5085766425192744

```
diUPy-k2co3-product-complex
   1.00000000000000
    25.3359200000000016    0.0000000000000000    0.0000000000000000
     0.0000000000000000   25.3596299999999992    0.0000000000000000
     0.0000000000000000    0.0000000000000000   21.8234199999999987
   C    H    N    O    K
    49    73     9    17     2
Direct
  0.3855722863151468  0.5883784910556118  0.6726221732885282
  0.4763011150947312  0.6012167208807900  0.6808084084723562
  0.4210709385320259  0.6774693503386986  0.6768646680115139
  0.3774359573713323  0.6456500490451076  0.6725520387877982
  0.4225314789757511  0.7365991347715196  0.6771494175648505
  0.3216143706315889  0.6656150396933834  0.6686986960420037
  0.2914951508265196  0.6518363000763659  0.6087864678506065
  0.3206783143652160  0.6679121804518414  0.5516363585410908
  0.3267161693858607  0.7298296172603552  0.4695120559901267
  0.2948083806807532  0.7751319303375093  0.4438151130290305
  0.5724365099023855  0.6128870364795638  0.6896903689837987
  0.6685132302896865  0.6110360399560506  0.6966765130159390
  0.6937672045639094  0.6193945714427191  0.6337216193670959
  0.5858596612064230  0.4472071101017428  0.6740336689148891
  0.4955280874466989  0.4334704224909177  0.6637088711654329
  0.5527548725192131  0.3596161962484255  0.6494237663591250
  0.5954728732482409  0.3913918621677429  0.6608325131426068
  0.5530925693012326  0.3021837346840198  0.6333422884692463
  0.6518561944723889  0.3728113064378794  0.6616027190565016
  0.6864999231597885  0.3945358399446314  0.6086541730400188
  0.6653746969609431  0.3801931085157919  0.5462962997186460
  0.6616419931521116  0.3140076249389726  0.4685656591505155
  0.6843583117116090  0.2597941918462280  0.4566716786569029
  0.3991636122538692  0.4198793070097270  0.6599007221724028
  0.3024439694867292  0.4203078968879761  0.6603258794979205
  0.2657602576898127  0.4485932388169557  0.6155858336383304
  0.4606901800928360  0.4989801237969240  0.4068803204156389
```

0.5097636102437105	0.3402338536784313	0.2743387493373804
0.5470550476867760	0.3758281186162149	0.2377525171665243
0.5232682572778465	0.4308725545570152	0.2256001248609996
0.5643422840834627	0.4752873386175987	0.2198371644324244
0.5858101093329021	0.4880097679580719	0.2830297684706806
0.6410065161393779	0.5493524664039147	0.3342977163877271
0.6747903048696410	0.5983191584459311	0.3280505378455950
0.6779282884031759	0.6305947003412050	0.3871813011048437
0.7135414612993599	0.6790576675774338	0.3813360157217391
0.7166675541785285	0.7117532153259299	0.4402204719944035
0.7520217400038460	0.7603498932417002	0.4341678608258662
0.4696519502656685	0.3180742526384810	0.3809886950926893
0.5165445940049136	0.3424811243616299	0.3432559258149664
0.3804286337182729	0.4201145234893683	0.4549316753653929
0.4255609063957908	0.3828248091625984	0.4594015754876575
0.4333017141626928	0.3606577667414609	0.4035782906359128
0.3941973306996807	0.3840486701842428	0.3602020857279672
0.3244942253730269	0.4546025817361360	0.3674197219424408
0.2677413364364434	0.4364141693002168	0.3792018817485143
0.2275748911868857	0.4740074370750195	0.3504578615411148
0.4918792621923014	0.2823427545903734	0.4308961981493869
0.4908675377999521	0.2238566874738528	0.4171708007041294
0.5061690643548866	0.6745448164135897	0.6853372662725866
0.4370607368578764	0.7512454546224890	0.7215443778065832
0.4499566038270358	0.7509412699744741	0.6417846250758225
0.3837856423541384	0.7540698046498964	0.6685833220403676
0.2987804413090192	0.6475362759713797	0.7061520860036480
0.3202971090965089	0.7084226397876373	0.6759416912877041
0.2865754851161890	0.6088542949098985	0.6071605433151086
0.2525401468687443	0.6707199394165740	0.6099767987229009
0.3673507928121992	0.7414782628137585	0.4810089266224825
0.3290357660319849	0.6962630799308029	0.4377716826737780
0.2928952171174526	0.8082231899605244	0.4762069979369346
0.3133648828943755	0.7892389849186705	0.4013544798629088
0.2543672770877576	0.7626663405720315	0.4329587494581000

0.6632479109485306	0.6490334675264853	0.7200683506076503
0.6940308847777641	0.5862079912971405	0.7254784011223897
0.6690678777585489	0.6451316773276129	0.6051532214088529
0.6989868417771165	0.5816797810812588	0.6096753527023090
0.5296562305409120	0.5404405130308096	0.6830138456235814
0.6165761653469642	0.5438031388036504	0.6909003278523503
0.7327359767469928	0.6380108701434863	0.6387015631557204
0.4676345065426782	0.3607609994115553	0.6463273814275773
0.5268231334130422	0.2800870122287876	0.6642284884631019
0.5383293514543765	0.2966305444571261	0.5862926058917546
0.5926279553466228	0.2850731018428432	0.6362000775593257
0.6702121201184017	0.3867856348998875	0.7043412456035233
0.6539681550702116	0.3295825630115700	0.6618571101887367
0.7266559726302823	0.3788115691609719	0.6138644514538075
0.6874900365255783	0.4376520273799709	0.6118596005658448
0.6182791181549745	0.3139209516490219	0.4699860721060458
0.6739318685218667	0.3427791399823282	0.4336665094989668
0.6719162243479364	0.2315850075185881	0.4920301332090521
0.6702725544580918	0.2453718959724814	0.4120821914533834
0.7276910293452106	0.2607523235576906	0.4555232000241721
0.3099164445894817	0.3796066035466338	0.6456230675718251
0.2841756212760750	0.4184697900424091	0.7062023059238811
0.2826798822580834	0.4486707474421724	0.5692655819832134
0.2589108561742546	0.4896173325078892	0.6298446422088769
0.4408158039784101	0.4922918386982889	0.6718577549525002
0.3538460380732851	0.4872741820549386	0.6694378746206938
0.2273301262215412	0.4284992308948320	0.6142056869717607
0.4678989365864447	0.3479616781976712	0.2638756878639758
0.5158223786370302	0.2980265103217024	0.2630430956329889
0.5568721562509819	0.3566454540744690	0.1939401219266406
0.5844197191419090	0.3790823340313747	0.2632143071546441
0.4992512339289062	0.4292494317982908	0.1836003930257083
0.5450949005554221	0.5107607432243639	0.2012412940970686
0.5963861396635358	0.4644756245634368	0.1879850097062686
0.6089544507502381	0.4550973874650395	0.3019932842918833

0.5526984260411657	0.4965941355511118	0.3142455496422726
0.7146119273815096	0.5850513713374299	0.3143431688880046
0.6598048091814266	0.6219770998702291	0.2895662674757422
0.6378315160199979	0.6430649714397068	0.4004331996851834
0.6920155052086855	0.6050151591779140	0.4245129684527659
0.7537102296690277	0.6665357092923546	0.3680381369288501
0.6992584891952844	0.7043647372337807	0.3436188302298678
0.6765274014286418	0.7240533293480982	0.4536305278247436
0.7311404528342380	0.6865388142653363	0.4778390417133718
0.7535606886607272	0.7829907637495201	0.4770998094610462
0.7927259906575702	0.7492015647741329	0.4222246609955909
0.7375720929444026	0.7869412911111715	0.3980103228166542
0.4470311989703252	0.2942205688659672	0.3479187826924872
0.4961759842078433	0.4416053889383479	0.2631076641758938
0.3313929171203707	0.4945207588098156	0.3855229891414765
0.2611661579270257	0.4335791476910730	0.4289638191315793
0.1869179087096641	0.4607291868190097	0.3588950747632190
0.2314223574826248	0.5140687613554323	0.3693493585867415
0.4456075267309714	0.3751267710255377	0.5023257481599969
0.3326405134568907	0.4558872619792473	0.3179582348022336
0.2626164880119439	0.3964457362383290	0.3603294851703117
0.2330372830141781	0.4768534042120134	0.3005382029938770
0.5114021935328821	0.2018173631305474	0.4531727949430744
0.4496672071840551	0.2102536512957444	0.4139544272739513
0.5090834917265119	0.2157766369152278	0.3723174013841281
0.4360994463350147	0.5680543081047217	0.6758875620198755
0.4700405237176146	0.6543972693873550	0.6817146837779932
0.5264740409897239	0.5816622706335728	0.6850774352541351
0.6172928863954185	0.5850933045504612	0.6933706664254884
0.5348934100370604	0.4666555245072401	0.6740950783814703
0.5030984047066376	0.3815180057161137	0.6517334210182802
0.4446394136804944	0.4517659333725568	0.6652449575405999
0.3535276554683249	0.4464745064450367	0.6651441641075450
0.3642516896166579	0.4201215996626203	0.3938544319350309
0.3007407713807089	0.7121499802077494	0.5257414491174109

0.3588180262782237	0.6441527583107619	0.5305236280883949
0.5702013706393374	0.6624423602249933	0.6906440989808010
0.3468895164154835	0.5572784474963514	0.6689451550683742
0.6814941595026091	0.3317358930958188	0.5280201195957518
0.6361165422501679	0.4081879690202928	0.5155364395030518
0.4025066238274738	0.3710080440277264	0.6518223444401847
0.6238885465418378	0.4777184608703571	0.6855523160149947
0.4697810941917558	0.4823938665366327	0.4641881011561605
0.4309319418188988	0.5394309821958717	0.3988275719634395
0.4824517622682473	0.4729779570659065	0.3624500692997614
0.6203916462093143	0.5342264845011028	0.2801345686638225
0.6335689493351736	0.5256787086995789	0.3827000348968214
0.5561238821670043	0.3592601373660048	0.3687959087994412
0.3582763219589979	0.4453487405399768	0.4961870042178162
0.3870338493665798	0.3728402851287467	0.3058717367687755
0.5106038305891369	0.2998054002106150	0.4785724027625842
0.3992370917516335	0.5439174611261057	0.5106918045500576
0.5644542122315522	0.4514343662858725	0.4367093606885985
