# Supplementary material for: Supramolecular interactions between catalytic species allow rational control over reaction kinetics
Source: Chem Sci. 2019 Aug 14;10(39):9115–24. doi: 10.1039/c9sc02357g (PMC6889839; doi:10.1039/c9sc02357g)
Supplement: Supplementary file 2 [file SC-010-C9SC02357G-s002.pdf]

Electronic Supplementary Material (ESI) for Chemical Science.
This journal is © The Royal Society of Chemistry 2019

Supporting information

Abraham J. P. Teunissen,[ab] Tim F. E. Paffen,[ab] Ivo A. W. Filot,[ac] Roy J. C. van der Haas,[ab] Menno D. Lanting,[ab] Tom F. A. de Greef*[ad] and E. W. Meijer*[ab]

[a] Institute for Complex Molecular Systems, Eindhoven University of Technology, P.O. Box 513, 5600 MB Eindhoven, The Netherlands; e-mail: e.w.meijer@tue.nl and t.a.f.d.greef@tue.nl.
[b] Laboratory of Macromolecular and Organic Chemistry, Eindhoven University of Technology, P.O. Box 513, 5600 MB Eindhoven, The Netherlands.
[c] Schuit institute for Catalysis, Eindhoven University of Technology, P.O. Box 513, 5600 MB Eindhoven, The Netherlands.
[d] Computational Biology, Eindhoven University of Technology, P.O. Box 513, 5600 MB Eindhoven, The Netherlands.

Table of contents

Materials and instrumentation S2
Determining the conversion of the Michael addition S3
Determining the association constant between NaPy 1 and UPy 2 .... S4
Synthetic procedures and Characterization S5
Synthesis of NaPy **1** S5
Synthesis of UPy **2** S8
Synthesis of $Pent_{ref}$ **4** S9
Synthesis of UPy_{pent} **5** S12
Synthesis of the Michael product S15
Kinetic computational modelling S17
References S23

Materials and Instrumentation

All used solvents were of analytical grade, all chemicals (including Mal_{ref} **3**) were purchased from Sigma Aldrich and used without further purification. Unless noted otherwise, all reactions were performed under argon, and all NMR measurements were performed in $CDCl_3$. All ^1H and ^{13}C NMR spectra were recorded on a Varian Mercury 400 MHz NMR or a 500 MHz Varian Unit Inova. Abbreviations used are s: singlet, d: doublet, d-d: double doublet, t: triplet, m: multiplet, b: broad. Deconvolution of NMR spectra was performed using MestReNova software version 7.1.1-9649. Data processing was performed using VNMRJ.3.2.a software. MALDI-TOF-MS was performed using a Perspective Biosystem Voyager-DE PRO spectrometer. Column chromatography was performed on a Biotage Isolera Spektra One Flash Chromatography system using KP-Sil Silica Gel SNAP columns. Immobilized *Candida Antarctica* Lipase B (Novozym 435) was obtained from Novozymes A/S and thoroughly dried under vacuum before use. The kinetic measurements on the Michael additions were performed on a 5 mL $CDCl_3$ scale in Wilmad screw-cap NMR tubes, diam. 10 mm, L 7 in. Solutions were made by mixing premade 20 mM stock solutions of all organic compounds. The tubes were shaken and rotated on a Hecht Assistant rotating mixer and removed for ≈ 20 minutes to measure their conversion by ^1H NMR. Conversions were determined by measuring the decrease in the signals associated with the maleimide and 2,4-pentanedione moieties. K_2CO_3 (99.995 % purity) was ground and filtered (< 0.125 mm) before use.

Simulations

Simulations were performed using the Matlab software package (R2016a, version 9.0.0341360, Mathworks) along with its optimization, curve fitting and symbolic math toolboxes. Where appropriate, mass balances were analytically solved using the Mathematica software package (version 9.0.1.0, Wolfram Research, Inc.). Otherwise, mass balances were solved numerically using either the *fzero* or *fsolve* function included in Matlab. Non-linear least squares optimizations were performed using the *lsqcurvefit* function from Matlabs optimization toolbox. This function uses the Levenberg-Marquardt method to minimize the residual sum of squares. A thousand fits were performed for each optimization. Initial parameters for the fits were distributed using latin hypercube sampling (implemented in the *lhsdesign* function), which ensures a uniform distribution in multidimensional parameterspace so that the global optimum can be obtained. The optimization with the lowest squared 2-norm is used as the best fit, while optimizations with a squared 2-norm within 5 percent of the best fit are considered equally good fits.

Determining the conversion of the Michael addition

^{1}H NMR was chosen to determine the conversion of the Michael addition, since this technique allows specific quantification of the various components of the reaction mixture at the millimolar regime in $CDCl_3$. Unfortunately, the signals corresponding to the Michael product still displayed much overlap with other signals (region 2.5-4.5 ppm). For this reason we chose to determine conversion by measuring the relative decrease of the signals associated with the unreacted propylmaleimide and 2,4-pentanedione compared to the TMS signal (Figure S1).

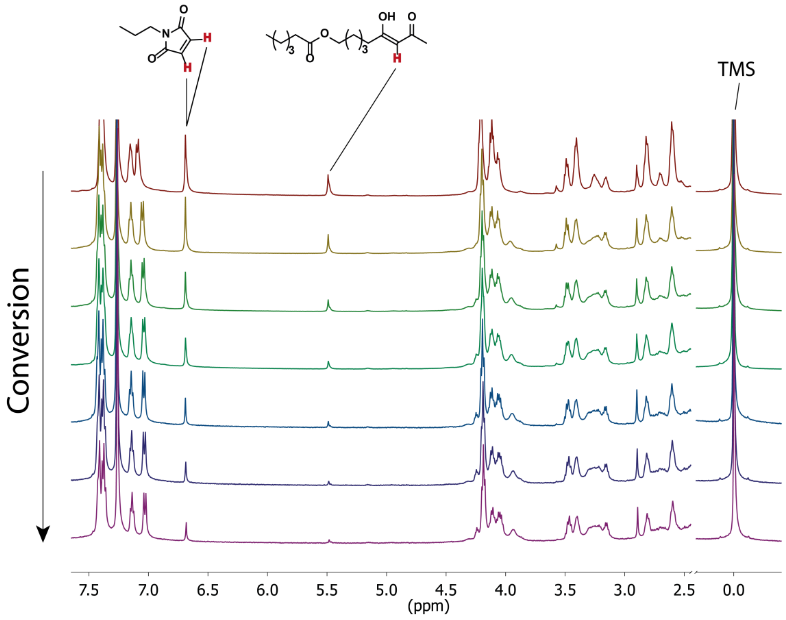

Figure S1: Typical ^{1}H NMR spectra of the Michael addition between Mal_{ref} **3** and $Pent_{ref}$ **4** in $CDCl_3$, depicting the protons used to determine the conversion.

Determining the association constant between NaPy 1 and UPy 2

In this project we wish to use the equilibrium between UPy and NaPy to regulate reaction kinetics. However, if the UPy-NaPy binding constant is too high, no UPy dimers will be present until more than one equivalent of UPy per NaPy is present. In that situation, UPy dimers and free NaPy will not be simultaneously present in significant amounts. The NaPy we used before has a UPy association constant of 6×10^7 M^{-1} in chloroform at 25 ˚C. This results in near quantitative binding of UPy, therefore making it NaPy unsuitable for this present study. After examining a library of NaPys we found that specific NaPy (Napy **1** see below) that is also catalytically active and has a significantly lower binding constant.

The equilibria of the UPy-NaPy system can be defined by equations 1-3. Since K_{dim} is know from literature (6×10^7 M^{-1} in chloroform at 25 ˚C) [1] and K_d can be determined by ^{1}H NMR, it is possible to determine K_a using equation 4.

$$K_{dim} = \frac{[U_2]}{[U]^2} \qquad \text{(eq. 1)}$$

$$K_d = \frac{[NU]^2}{[U_2]*[N]^2} \qquad \text{(eq. 2)}$$

$$K_a = \frac{[NU]^2}{[U]*[N]} \qquad \text{(eq. 3)}$$

$$K_a = \sqrt{(K_{dim} * K_d)} \qquad \text{(eq. 4)}$$

A sample containing NaPy **1** and UPy **2** (4 mM each) was prepared and analyzed by ^{1}H NMR in $CDCl_3$ (Figure S2). Signals corresponding to protons *a* and *b* were used to calculate K_a, resulting in a value of 5.3 +/- 0.2×10^5 M^{-1}.

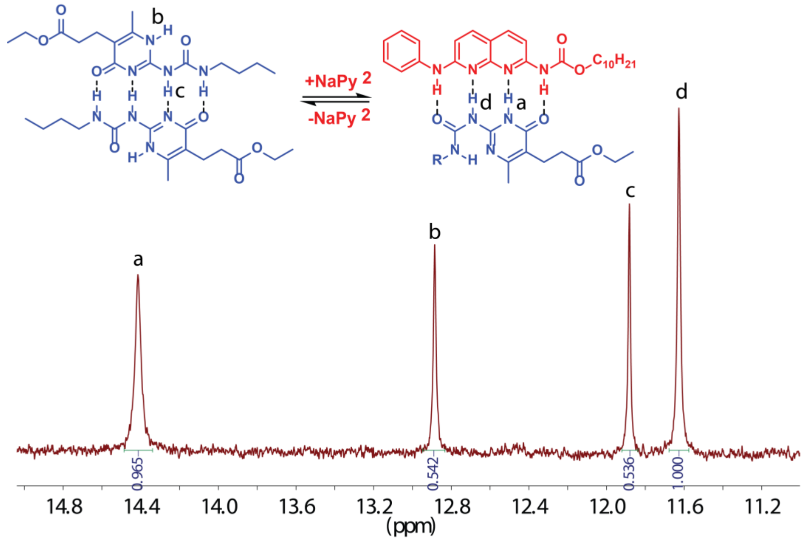

Figure S2: Molecular structure of the UPy-UPy and UPy-NaPy dimers and the corresponding ^{1}H NMR signals in a mixture of UPy and NaPy (4 mM each in $CDCl_3$ at 25˚C) used to determine K_a.

Synthetic procedures and characterization

Synthesis of NaPy 1

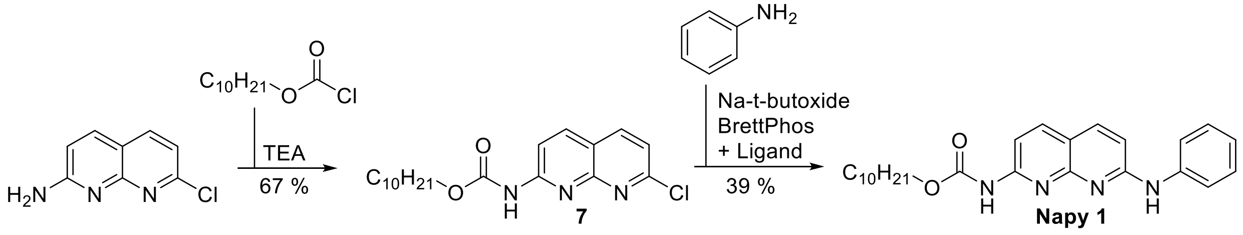

Scheme S1: Synthetic scheme of NaPy **1**.

decyl (7-chloro-1,8-naphthyridin-2-yl)carbamate (7)

7-chloro-1,8-naphthyridin-2-amine (2.30 g, 12.8 mmol), decyl carbonochloridate (4.50 g, 20.4 mmol) and trimethylamine (3.88 g, 38.3 mmol) were dissolved in chloroform (30 mL) and stirred for 16 hours at 40 ˚C. Chloroform (30 mL) was added and the mixture was washed with water (3 x 30 mL). The crude product was purified by column chromatography (0 - 25 % EtOAc in heptanes). Yield = 3.12 g, 8.58 mmol. η = 67 %. ^{1}H NMR (400 MHz, $CDCl_3$) δ = 8.35 (d, 1H, Ar-H), 8.15 (d, 1H, Ar-H), 8.04 (d, 1H, Ar-H), 7.72 (b, 1H, N-H), 7.37 (d, 1H, Ar-H), 4.23 (t, 2H, C=O-CH, 1.71 (p, 2H, CH_2), 1.27 (m, 14H, CH_2), 0.88 (t, 3H, CH_3). ^{13}C NMR (100 MHz, $CDCl_3$) δ: 154.64, 154.54, 154.42, 153.27, 139.14, 138.92, 121.95, 118.80, 113.89, 66.42, 32.03, 29.66, 29.45, 29.38, 28.88, 25.93, 22.83, 14.27. MALDI-ToF MS: calculated 363.88, observed 364.29 [$M+H^+$] and 386.26 [$M+Na^+$].

decyl (7-(phenylamino)-1,8-naphthyridin-2-yl)carbamate (NaPy 1)

decyl (7-chloro-1,8-naphthyridin-2-yl)carbamate **7** (200 mg 0.55 mmol), sodiumtertbutoxide (60 mg, 0.63 mmol), BrettPhos (36 mg) and BrettPhos Pd G1 Methyl-t-butyl ether adduct (43 mg) were combined in a shlenk tube capped with a septum. The flask was evacuated and backfilled with argon 3 times. Aniline (72 mg, 0.77 mmol) in dry dibutyl ether (12 mL) was added and the shlenk tube was placed in an oil bath at 95 ˚C. The mixture was stirred at this temperature and under argon for 1 hour after which it was allowed to cool to room temperature. The crude product was diluted with EtOAc (50 mL) and was with water (3 x 30 mL). The organic phase was dried thoroughly under vacuum and purified using column chromatography (10 - 30 % EtOAc in heptanes). Yield = 90 mg, 0.21 mmol. η = 39 %. ^{1}H NMR (400 MHz, $CDCl_3$) δ = 8.01 (d, 1H, J = 8.6 Hz, Ar-H), 7.93 (d, 1H, J = 8.6 Hz, Ar-H), 7.81 (d, 1H, J = 8.8 Hz, Ar-H), 7.57 (bs, 2H, NH, Ar-H), 7.53 (d, 2H, J = 8.2 Hz, Ar-H), 7.38 (t, 2H, J = 7.7 Hz, Ar-H), 7.14 (t, 1H, J = 7.4 Hz, Ar-H), 6.91 (d, 1H, J = 8.7 Hz, Ar-H), 4.19 (t, 2H, J = 6.7 Hz, O-CH_2), 1.69 (p, 2H, J = 6.8 Hz, O-CH_2-C$\underline{H_2}$), 1.40 – 1.27 (m, 14H, CH_2), 0.87 (t, J = 6.7 Hz, 3H, CH_3). ^{13}C NMR (100 MHz, $CDCl_3$) δ: 157.85, 155.55, 153.57, 153.46, 139.48, 138.71, 137.85, 129.49, 124.27, 122.17, 115.30, 110.20, 109.31, 65.97, 32.03, 29.67, 29.45, 29.39, 28.94, 25.96, 22.83, 14.27. MALDI-ToF MS: calculated 420.55, observed 421.29 [$M+H^+$].

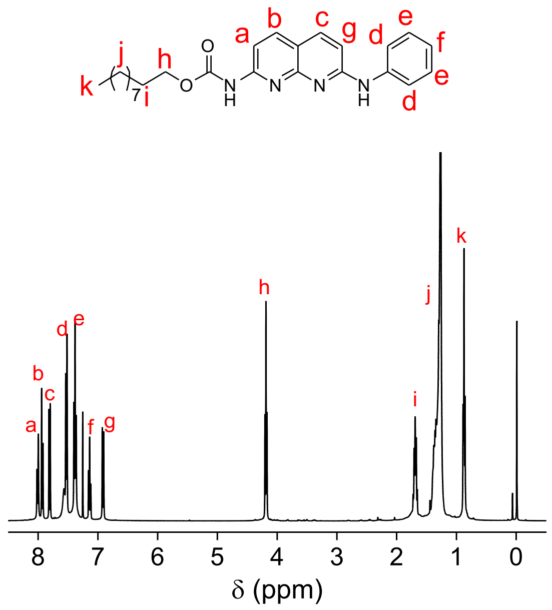

Figure S3: ^{1}H NMR spectrum of NaPy **1** in $CDCl_3$.

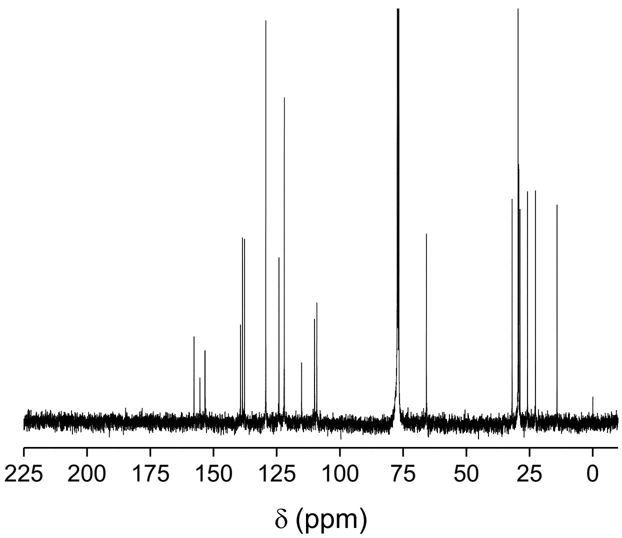

Figure S4: ^{13}C NMR spectrum of NaPy **1** in $CDCl_3$.

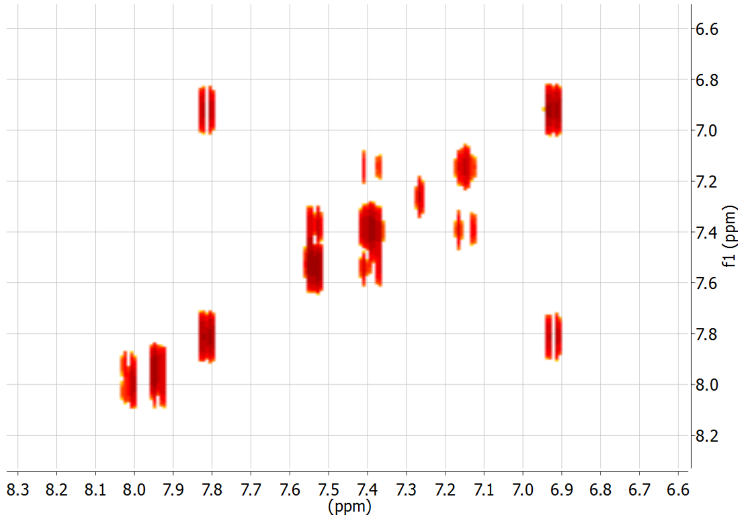

Figure S5: COSY NMR spectrum of NaPy **1** in $CDCl_3$.

Synthesis of UPy 2

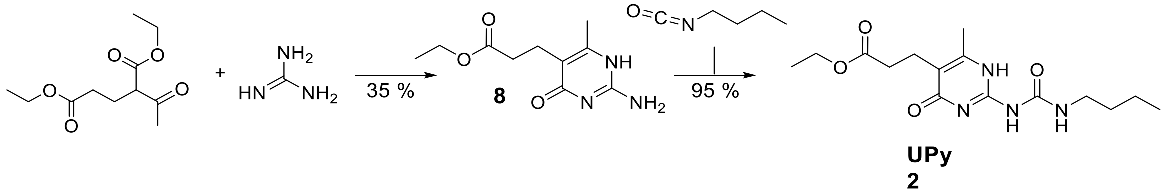

Scheme S2: Synthetic scheme of UPy **2**.

Ethyl 3-(2-amino-6-methyl-4-oxo-1,4-dihydropyrimidin-5-yl)propanoate (8)

A mixture of diethyl 2-acetylpentanedioate (1.86 mL, 8.69 mmol) and guanidine carbonate (784 mg, 8.69 mmol) in ethanol (20 mL) was stirred for 18 hours under reflux. The reaction mixture was allowed to cool to room temperature. Precipitation at 0 °C yielded the product as a white solid. Yield = 690 mg, 3.07 mmol. η = 35 %. ^{1}H NMR (400 MHz, $CDCl_3$) δ: 4.09 (q, 2H, O-C$\underline{H}_2$-CH_3), 2.70 (t, 2H, O=C-C$\underline{H}_2$-CH_2), 2.51 (t, 2H, O=C-CH_2-C$\underline{H}_2$), 2.21 (s, 3H, C-CH_3), 1.26 (t, 3H, CH_2-C$\underline{H}_3$). ^{13}C NMR (100 MHz, d-DMSO) δ = 172.61, 153.45, 109.33, 79.19, 59.72, 32.74, 20.96, 14.13. MALDI-ToF MS: calculated 225.24, observed m/z 226.29 [$M+H^+$], 248.24 [$M+Na^+$].

Ethyl 3-(2-(3-butylureido)-6-methyl-4-oxo-1,4-dihydropyrimidin-5-yl)propanoate (UPy 2)

Ethyl 3-(2-amino-6-methyl-4-oxo-1,4-dihydropyrimidin-5-yl)propanoate **8** (1.03 g, 4.57 mmol) in dry DMF (25 mL) was heated to 70 °C. 1-Isocyanatobutane (770 µL, 6.86 mmol) was added and the reaction mixture was stirred for 6 hours at 70 °C. The product was obtained as a white solid after evaporation of the solvent *in vacuo*. Yield = 1.41 g, 4.35 mmol. η = 95 %. ^{1}H NMR (400 MHz, $CDCl_3$) δ: 12.88 (bs, 1H, NH), 11.89 (bs, 1H, NH), 10.11 (bs, 1H, NH), 4.08 (q, 2H, O-C$\underline{H}_2$-CH_3), 3.24 (q, 2H, NH-(C=O)-NH-C$\underline{H}_2$), 2.68 (t, 2H, O-(C=O)-C$\underline{H}_2$-CH_2), 2.60 (t, 2H, O-(C=O)-CH_2-C$\underline{H}_2$), 2.30 (s, 3H, C-CH_3), 1.57 (t, 2H, alkyl-CH_2), 1.41 (t, 2H, CH_2-C$\underline{H}_2$-CH_3), 1.24 (t, 3H, O-CH_2-C$\underline{H}_3$), 0.94 (t, 3H, C$\underline{H}_3$). ^{13}C NMR (100 MHz, $CDCl_3$): 173.32, 156.64, 153.31, 143.97, 116.09, 104.99, 60.34, 39.66, 32.12, 31.35, 21.15, 20.12, 17.17, 14.23, 13.75. MALDI-ToF MS: calculated 324.38, observed m/z 325.30 [$M+H^+$], 347.27 [$M+Na^+$].

Synthesis of Pent$_{ref}$ 4

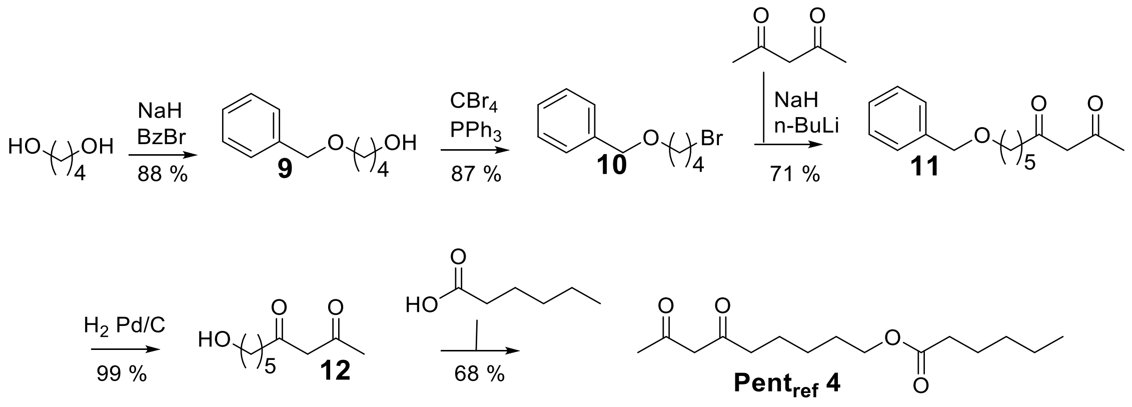

Scheme S3: Synthetic scheme of Pent$_{ref}$ **4**.

4-(Benzyloxy)butan-1-ol (9)

Dry DMF (400 mL) was added to NaH (60 % dispersion in oil, 1.95 g, 49.0 mmol and cooled to 0 °C. Butane-1,4-diol (4.00 g, 44.0 mmol) in dry DMF (20 mL) was added slowly and the reaction mixture was stirred for 10 minutes. Benzyl bromide (5.31 mL, 44.4 mmol) was added to the mixture cautiously. The mixture was allowed to cool acclimate to room temperature and stirred for 18 hours. Subsequently, the reaction was quenched upon the addition of H_2O (90 mL) and extracted with EtOAc (5 x 100 mL). The organic layer was dried with $MgSO_4$ and filtered. The filtrate was concentrated by evaporation of the solvent *in vacuo*. The product was obtained as colorless oil. Yield = 6.88 g, 38 mmol. ɳ = 88 %. ^{1}H NMR (400 MHz, $CDCl_3$) δ: 7.32 (m, 5H, Ar), 4.50 (s, 2H, Ar-C$\underline{H}_2$-O), 3.60 (t, 2H, CH_2-C$\underline{H}_2$-OH), 3.50 (t, 2H, O-C$\underline{H}_2$-CH_2), 1.66 (m, 4H, alkyl-CH_2). ^{13}C NMR (100 MHz, $CDCl_3$): 138.14, 128.40, 127.70, 127.64, 73.03, 70.32, 62.61, 30.07, 26.64.

((4-Bromobutoxy)methyl)benzene (10)

CBr_4 (3.75 g, 11.3 mmol) was added to a solution of 4-(benzyloxy)butan-1-ol **9** (1.69 g, 9.4 mmol) in dry CH_2Cl_2 (30 mL) and cooled to 0 °C. PPh_3 (4.93 mmol, 18.8 mmol) was added in portions. The resulting solution was stirred for 16 hours at room temperature and concentrated *in vacuo*. The remaining solids were washed with ether (5 x 50 mL) and filtrated; followed by concentration of the collective ether extracts *in vacuo*. Purification using column chromatograph (96 % Heptane / 4 % EtOAc) yielded the target compound. Yield = 1.98 g, 8.18 mmol. ɳ = 87 %. ^{1}H NMR (400 MHz, $CDCl_3$) δ: 7.33 (m, 5H, Ar), 4.50 (s, 2H, Ar-C$\underline{H}_2$-O), 3.50 (t, 2H, O-C$\underline{H}_2$-CH_2), 3.43 (t, 2H, CH_2-C$\underline{H}_2$-Br), 1.98 (m, 2H, alkyl-CH_2), 1.76 (m, 2H, alkyl-CH_2). ^{13}C NMR (100 MHz, $CDCl_3$) δ: 138.68, 128.62, 127.83, 127.81, 73.15, 69.47, 33.99, 29.96, 28.60.

9-(Benzyloxy)nonane-2,4-dione (11)

2,4-Pentanedione (1.31 g, 13.1 mmol) was slowly added to a suspension of NaH (60 % dispersion in oil) (522 mg, 13.1 mmol) in THF (25 mL) cooled to 0 °C. The mixture was stirred for 10 minutes. n-BuLi (1.6 M in hexanes, 6.56 mL) was added dropwise over a period of 15 minutes. The mixture was stirred for 20 minutes at 0 °C, ((4-bromobutoxy)methyl)benzene **10** (2.79 g, 11.5 mmol) in THF (1.5 mL) was added dropwise and the mixture was stirred for 2 hours at room temperature. The reaction was quenched with a mixture of concentrated HCl (2 mL) in H_2O (2.5 mL) and Et_2O (10 mL). Subsequently, the

organic layer was washed with brine (3 x 20 mL) and dried with $MgSO_4$. After filtration, the filtrate was concentrated *in vacuo*. The crude product was purified using column chromatography (93 % Heptane / 7 % EtOAc). Yield = 2.13 g, 8.14 mmol. η = 71 %. ^{1}H NMR (400 MHz, $CDCl_3$) δ: 7.32 (m, 5H, Ar), 5.47 (s, 1H, ((C=O)-CH=COH), 4.48 (s, 2H, Ar-CH_2-O), 3.53 (s, 2H, (C=O)-CH_2-(C=O)), 3.46 (t, 2H, O-CH_2-CH_2), 2.26 (t, 2H, CH_2-CH_2-C=O), 2.20 (s, 3H, CH_2-(C=O)-CH_3), 2.03 (s, 3H, CH=COH-CH_3), 1.62 (m, 4H, alkyl-CH_2), 1.40 (m, 2H, alkyl-CH_2). ^{13}C NMR (100 MHz, $CDCl_3$) δ: 204.06, 202.11, 194.02, 191.42, 138.56, 128.33, 127.60, 127.49, 127.48, 99.77, 72.89, 70.11, 70.05, 43.70, 38.15, 31.87, 30.88, 29.69, 29.47, 25.86, 25.65, 25.49, 24.97, 24.96, 23.15, 22.68, 14.11. MALDI-ToF MS: calculated 262.34, observed 262.26 [$M+H^+$], 285.20 [$M+Na^+$], 301.16 [$M+K^+$].

9-Hydroxynonane-2,4-dione (12)

9-(Benzyloxy)nonane-2,4-dione **11** (2.12 g, 8.10 mmol) in EtOAc (30 mL) was bubbled through with N_2 for 10 minutes. A spatula tip Pd/C was added to the mixture and the reaction vessel was placed under H_2-atmosphere and shaken in a Parr-reactor (70 psi, 18 h). Filtration over Celite and subsequent concentration *in vacuo*, yielded the product. Yield = 1.39 g, 8.04 mmol. η = 99 %. ^{1}H NMR (100 MHz, $CDCl_3$) δ: 5.49 (s, 1H, ((C=O)-CH=COH), 3.66 (t, 2H, CH_2-CH_2-OH), 3.57 (s, 2H, (C=O)-CH_2-(C=O)), 2.29 (t, 2H, CH_2-CH_2-C=O), 2.24 (s, 3H, CH_2-(C=O)-CH_3), 2.05 (s, 3H, CH=COH-CH_3), 1.59 (m, 4H, alkyl-CH_2), 1.41 (m, 2H, alkyl-CH_2). ^{13}C NMR (100 MHz, $CDCl_3$) δ: 204.25, 202.35, 194.17, 191.35, 62.52, 62.42, 57.81, 43.66, 38.16, 25.36, 25.11, 24.91, 22.97, 14.11. MALDI-ToF MS: calculated 172.11, observed m/z 173.24 [$M+H^+$], 195.19 [$M+Na^+$].

6,8-Dioxononyl hexanoate ($Pent_{ref}$ 4)

9-Hydroxynonane-2,4-dione **12** (159 mg, 0.92 mmol) and hexanoic acid (115.6 µl, 0.92 mmol) were dissolved in toluene (150 mL) and Novozym 435 (64 mg) was added to the reaction mixture. The reaction mixture was slowly stirred using a rotovap (65 °C, 250 mbar) for 8 hours, followed by filtration and concentration *in vacuo*. Purification by column chromatograph (88 % $CHCl_3$ / 12 % methanol) yielded the product. Yield = 169 mg, 0.62 mmol. η = 68 %. ^{1}H NMR (100 MHz, $CDCl_3$) δ: 5.48 (s, 1H, ((C=O)-CH=COH), 4.06 (t, 2H, O=C-O-CH_2), 3.57 (s, 2H, (C=O)-CH_2-(C=O)), 2.29 (t, 2H, CH_2-CH_2-C=O), 2.29 (t, 2H, O-(C=O)-CH_2), 2.24 (s, 3H, CH_2-(C=O)-CH_3), 2.05 (s, 3H, CH=COH-CH_3), 1.64-1.31 (m, 12H, alkyl-CH_2), 0.89 (t, 3H, alkyl-CH_3). ^{13}C NMR (100 MHz, $CDCl_3$) δ: 193.85, 191.36, 173.93, 99.76, 63.98, 38.06, 34.32, 31.30, 28.40, 25.58, 25.24, 24.94, 24.67, 22.30, 13.90. MALDI-ToF MS: calculated 270.36, observed m/z 293.26 [$M+Na^+$].

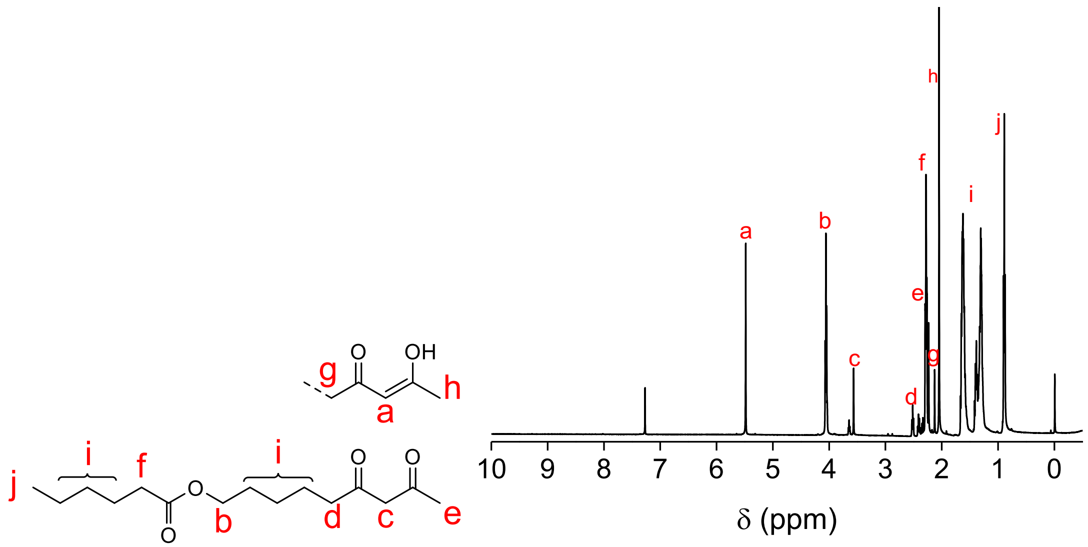

Figure S6: ^{1}H NMR spectrum of Pent$_{ref}$ **4** in $CDCl_3$.

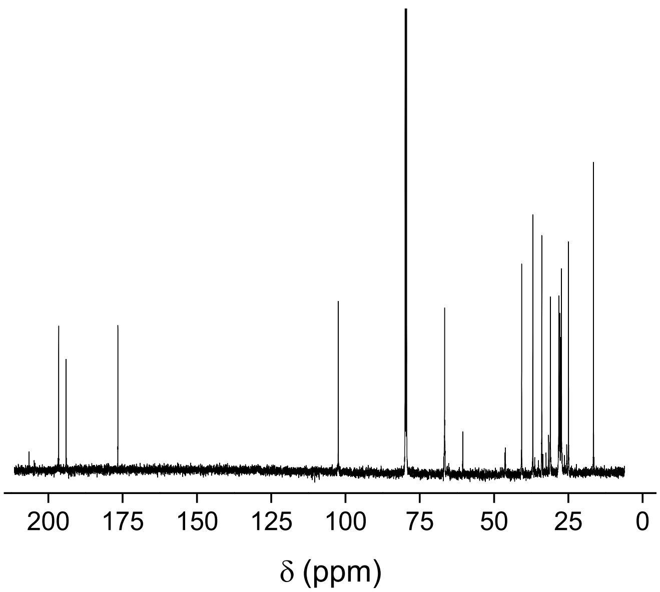

Figure S7: ^{13}C NMR spectrum of Pent$_{ref}$ **4** in $CDCl_3$.

Synthesis of UPy$_{pent}$ 5

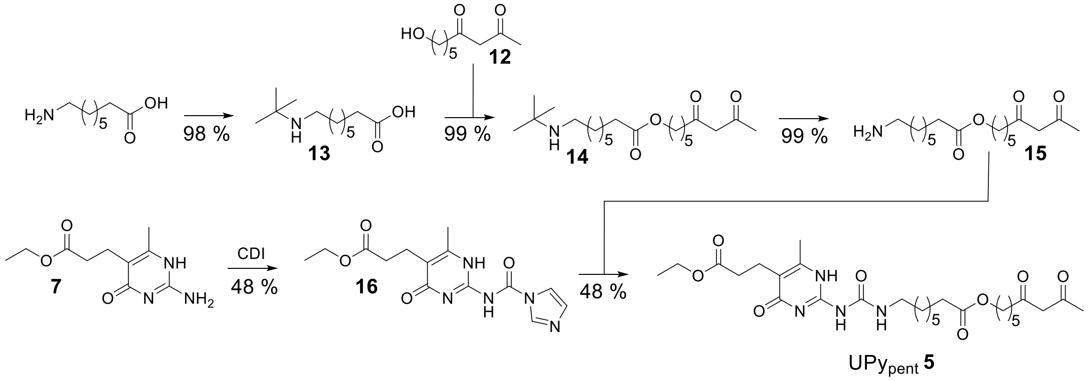

Scheme S4: Synthetic scheme of UPy$_{pent}$ **5**.

8-((Tert-butoxycarbonyl)amino)octanoic acid (13)

8-aminooctanoic acid (3.0 g, 18.8 mmol) was dissolved in a mixture of H_2O (15mL) and DCM (15 mL), NaOH (1.51 g, 37.7 mmol) was added and the mixture was cooled to 0 ˚C. Di-tert-butyl dicarbonate (4.11, 18.8 mmol) in DCM (40 mL) was slowly added. The mixture was stirred at room temperature for 24 hours, conc. HCl (3 mL) was added and the aqueous phase was extracted with DCM (2 x 30 mL). The organic phases were dried using $MgSO_4$, filtered and dried under vacuum to yield the target compound. Yield = 4.78 g, 18.4 mmol. ɳ = 98 %. ^{1}H NMR (400 MHz, $CDCl_3$) δ: 4.53 (b, 1H, NH), 3.09 (m, 2H, CH_2-NH), 2.34 (t, 2H, *J* = 7.5 Hz, CH_2-C=O), 1.63 (p, 2H, *j* = 7.1 Hz, CH_2), 1,44 (m, 11H, CH_2+CH_3), 1.32 (m, 6H, CH_2). ^{13}C NMR (100 MHz, $CDCl_3$) δ: 179.17, 156.03, 79.11, 40.56, 33.98, 29.96, 28.95, 28.43, 26.56, 24.61. FT-IR ν~= 3367, 2977, 2936, 2858, 1685, 1523, 1173 cm^{-1}.

6,8-Dioxononyl 8-((tert-butoxycarbonyl)amino)octanoate (14)

8-((tert-butoxycarbonyl)amino)octanoic acid **13** (150 mg, 0.57 mmol) and 9-Hydroxynonane-2,4-dione **11** were dissolved (100 mg, 0.57 mmol) were dissolved in toluene (15 mL), a spatula tip of Novozyme 435 was added and the mixture was stirred at 50 ˚C and 250 mbar on a rotavapor for 8 hours. The Novozyme beads were filtered off and the solvent evaporated to yield the product. Yield = 236 mg, 0.57 mmol. ɳ = >99 %. ^{1}H NMR (400 MHz, $CDCl_3$) δ: 5.49 (s, 1H, CH-COH), 4.52 (b, 1H, NH), 4.06 (m, 2H, O-CH_2), 3.57 (s, 2H, C=O-CH_2), 3.11 (q, 2H, NH-CH_2, J = 6.1 Hz), 2.52 (t, 2H, CH_2-enol, J= 7.3 Hz), 2.28 (t, 2H, CH_2-keto + C=O-CH_2, J= 7.5 Hz), 2.24 (s, 3H, CH_3-keto), 2.06 (s, 3H, CH_3-enol), 1.68-1.53 (m, 8H, CH_2), 1.44 (s, 9H, CH_3), 1.25-1.36 (m, 8H, CH_2). ^{13}C NMR (100 MHz, $CDCl_3$) δ: 193.90, 191.37, 173.86, 99.80, 64.04, 40.59, 38.09, 34.29, 30.01, 29.05, 28.92, 28.42, 26.61, 25.60, 25.26, 24.96, 24.88.

8-((6,8-Dioxononyl)oxy)-8-oxooctan-1-aminium chloride (15)

6,8-dioxononyl 8-((tert-butoxycarbonyl)amino)octanoate **14** (230 mg, 0.56 mmol) was dissolved in 3 M HCl in dioxane (20 mL) and stirred for 4 hours. The solvent as evaporated to yield the product. Yield = 194 mg, 0.55 mmol). ɳ = >99 %. ^{1}H NMR (400 MHz, $CDCl_3$) δ: 8.28 (b, 3H, NH_3^+), 5.49 (s, 1H, C=O-CH-C=O), 4.05 (t, 2H, O-CH_2), 2.98 (b, 2H, NH_3^+-CH_2), 2.27 (dt, 4H, O-C=O-CH_2 + C=O-CH_2), 2.05 (s, 3H, CH=COH-CH_3), 1.87-1.25 (m, 16, CH_2). ^{13}C NMR (100 MHz, $CDCl_3$) δ: 193.92, 191.41, 173.87, 99.83, 64.13, 39.95, 38.08, 34.19, 28.80, 28.59, 28.40, 27.54, 26.25, 25.59, 25.26, 24.98, 24.73. MALDI-ToF MS: calculated 349.20, observed m/z 314.22 [M-Cl$^-$].

Ethyl 3-(2-(1H-imidazole-1-carboxamido)-6-methyl-4-oxo-1,4-dihydropyrimidin-5-yl)propanoate (16)
ethyl 3-(2-amino-6-methyl-4-oxo-1,4-dihydropyrimidin-5-yl)propanoate **7** (1.0 g, 4.44 mmol) was suspended in chloroform (80 mL) and CDI (800 mg, 4.94 mmol) was added. The mixture was refluxed overnight under Argon overnight and subsequently allowed to cool to room temperature. The solution was washed with water (5 x 100 mL) and dried using $MgSO_4$ to afford the product. Yield = 462 mg, 1.45 mmol). η = 33 %. ^{1}H NMR (400 MHz, $CDCl_3$) δ: 13.61 (b, 1H, NH), 12.38 (b, 1H, NH), 8.85 (s, 1H, N=CH), 7.61 (s, 1H, CH=CH), 7.02 (s, 1H, CH=CH), 4.12 (q, 2H, J = 7.1 Hz, O-CH_2), 2.77 (t, 2H, J = 7.1 Hz, C=O-C$\underline{H_2}$-CH_2), 2.63 (t, 2H, J = 7.1 Hz, C=O-CH_2-C$\underline{H_2}$), 2.50 (s, 3H, CH_3), 1.26 (t, 3H, J = 7.1 Hz, CH_3). ^{13}C NMR (100 MHz, $CDCl_3$) δ 172.89, 160.85, 157.00, 155.28, 148.37, 137.99, 127.66, 117.55, 114.84, 60.58, 32.13, 20.79, 16.56, 14.23. FT-IR (ATR) $\tilde{\nu}$ = 3163, 2980, 2931, 2719, 1726, 1681, 1642, 1610, 1472, 1671, 1323, 1220, 1183, 1094, 1064, 1005, 860.

6,8-Dioxononyl 8-(3-(5-(3-ethoxy-3-oxopropyl)-6-methyl-4-oxo-1,4-dihydropyrimidin-2-yl)ureido) octanoate (UPy$_{pent}$ 5)
ethyl 3-(2-(1H-imidazole-1-carboxamido)-6-methyl-4-oxo-1,4-dihydropyrimidin-5-yl)propanoate **16** (208 mg, 0.65 mmol) and trimethylamine (66 mg, 0.65 mmol) were dissolved in chloroform (30 mL) and a solution of 8-((6,8-dioxononyl)oxy)-8-oxooctan-1-aminium chloride **15** (190 mg, 0.54 mmol) chloroform (5 mL) was slowly added. The mixture was stirred overnight at room temperature, chloroform (30 mL) was added and the mixture was washed with water (3 x 30 mL). The organic phase was dried using $MgSO_4$ and purified using column chromatography (0 - 5 % MeOH in chloroform), followed by a second purification using column chromatography (0 - 30 % EtOAc in Heptanes). Yield = 148 mg, 0.26 mmol. η = 48 %. ^{1}H NMR (400 MHz, $CDCl_3$) δ: 12.89 (b, 1H, NH), 11.88 (b, 1H, NH), 10.14 (b, 1H, NH), 5.48 (C=O-C$\underline{H}$=COH), 4.09 (q, 2H, J = 7.2 Hz, O-C$\underline{H_2}$-CH_3), 4.05 (t, 2H, J = 6.6 Hz, O-C$\underline{H_2}$-CH_2), 3.57 (s, 2H, C=O-CH_2-C=O), 3.23 (q, 2H, J = 6.7 Hz, NH-C$\underline{H_2}$), 2.70 (t, 2H, J = 6.9 Hz, C=O-C$\underline{H_2}$-CH_2), 2.59 (t, 2H, J = 7.0 Hz, C=O-CH_2-C$\underline{H_2}$), 2.52 (t, 2H, J = 7.3 Hz, C$\underline{H_2}$-C=O), 2.30 (s, 3H, CH_3), 2.28 (dt and s, 7H, O-C=O-CH_2 + C=O-CH_2, C=O-CH_3), 2.24 (s, 3H, CH_2-C=O-C$\underline{H_3}$) 2.05 (s, 3H, CH=COH-CH_3), 1.62 (m, 8H, CH_2), 1.34 (m, 8H, CH_2), 1.24 (t, 3H, J = 7.1 Hz, CH_2-CH_3). ^{13}C NMR (100 MHz, $CDCl_3$) δ: 194.02, 191.51, 174.01, 173.45, 172.26, 156.83, 153.49, 144.15, 116.26, 99.94, 64.15, 60.50, 43.70, 40.07, 38.22, 34.45, 32.29, 29.43, 29.25, 29.12, 28.55, 26.96, 25.72, 25.39, 25.10, 21.28, 17.35, 14.40. MALDI-ToF MS: calculated 564.71, observed m/z: 565.35 [M+H$^+$].

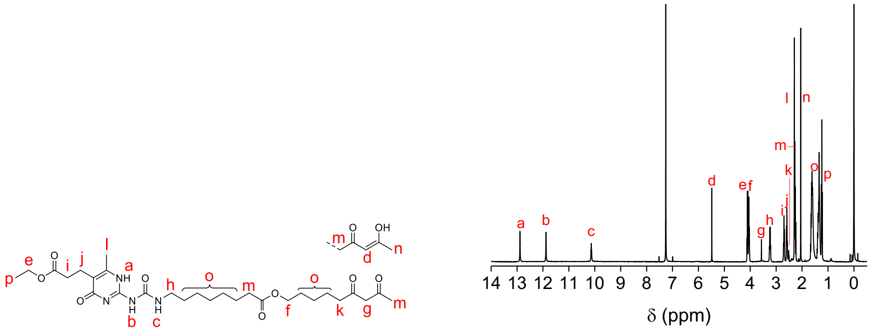

Figure S8: ^{1}H NMR spectrum of UPy$_{pent}$ **5** in $CDCl_3$.

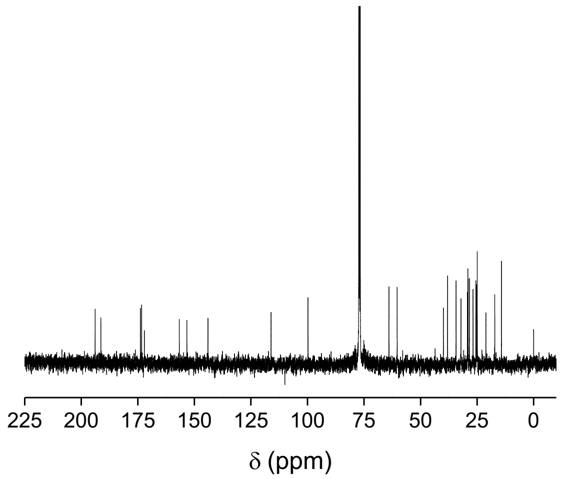

Figure S9: ^{13}C NMR spectrum of UPy_{pen} **5** in $CDCl_3$.

Synthesis of the Michael product (6)

Mal_{ref} **3** (67 mg, 0.49 mmol) and $Pent_{ref}$ **4** (131 mg, 0,49 mmol) were dissolved in $CDCl_3$ (4 mL), K_2CO_3 (167 mg, 1.21 mmol) and 18-crown-6 (64 mg, 0.24 mmol) were added. The mixture was stirred for 2 hours, the K_2CO_3 was filtered off, chloroform (20 mL) was added and the organic phase was with water (5 x 20 mL). The crude product was purified using column chromatography twice (0 - 5 % MeOH in chloroform). Yield = 97 mg, η = 49 %. ^1H NMR (400 MHz, $CDCl_3$) δ: 4.26 (m, 1H, C=O-CH), 4.07 (m, 2H, CH_2-C=O), 3.47 (t, 2H, N-CH_2), 3.29 (m, 1H, CH_2-C<u>H</u>), 2.42-2.80 (m, 4H, CH-C<u>H₂</u> + C=O-CH_2), 2.34 (s, 3H, CH_3), 2.28 (m, 2H, O-CH_2), 2.18 (s, 3H, CH_3), 1.55-1.72 (m, 8H, CH_2), 1.26-1.42 (m, 6H, CH_2), 0.90 (m, 6H, CH_3). ^{13}C NMR (101 MHz, $CDCl_3$) δ: 204.60, 204.15, 202.56, 201.83, 177.84, 175.67, 173.92, 65.10, 65.05, 63.87, 63.84, 43.13, 42.84, 40.70, 39.08, 39.01, 34.30, 32.16, 32.12, 31.32, 30.13, 29.85, 28.46, 28.41, 25.39, 25.30, 24.67, 22.98, 22.91, 22.31, 20.86, 13.91, 11.23. MALDI-ToF MS: calculated 409.53, observed 432.26 [M+Na^+] and 571.32 [M+K_2CO_3+Na^+].

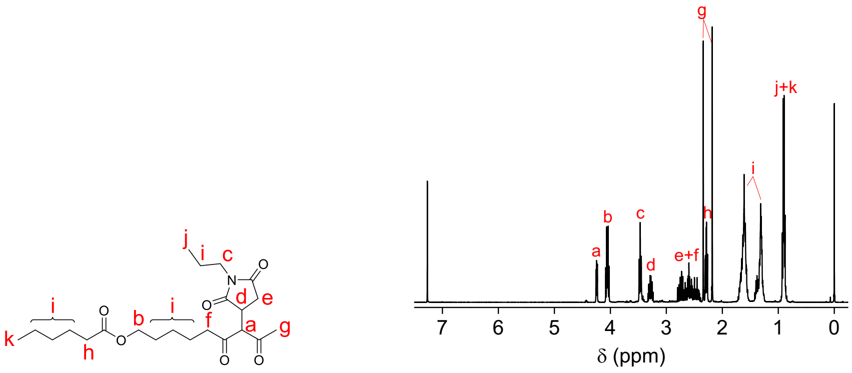

Figure S10: ^1H NMR spectrum of the Michael product **6** in $CDCl_3$.

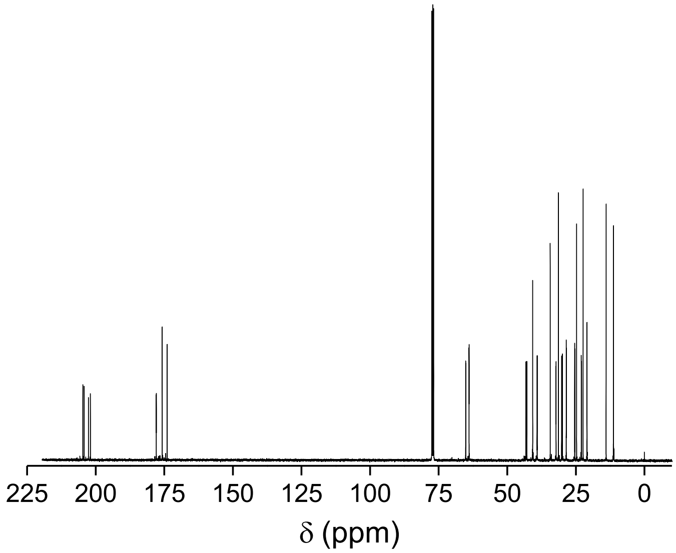

Figure S11: ^{13}C NMR spectrum of the Michael product **6** in $CDCl_3$.

Kinetic modelling

Kinetic model for the Michael addition of Mal$_{ref}$ ***3*** *and Pent$_{ref}$* ***4***

For the experiments where Mal$_{ref}$ **3** and Pent$_{ref}$ **4** are used as reagents, the overall reaction rate of the Michael addition is described by mass action kinetics, *i.e.* the rate of the reaction, r_{M1}, is directly proportional to the concentrations of reagents and catalysts (Eq S1). The only exception to this is the catalysis by free NaPy and UPy-NaPy heterodimers since the experimental data could not be described with reaction orders of unity. Therefore, the reaction orders were added as free parameters.

$$r_{M1} = \left[\mathrm{Mal_{ref}}\right]\cdot\left[\mathrm{Pent_{ref}}\right]\cdot\begin{pmatrix} k_{\mathrm{K_2CO_3}}\left[\mathrm{K_2CO_3}\right]+k_{\mathrm{P}}\left[\mathrm{P}\right] \\ +k_{\mathrm{U_2}}\left[\mathrm{U_{2c}}\right]+k_{\mathrm{U_2P}}\left[\mathrm{U_2P_c}\right] \\ +k_{\mathrm{UN}}\left[\mathrm{UN}\right]^{a_{\mathrm{UN}}}+k_{\mathrm{N}}\left[\mathrm{N}\right]^{a_{\mathrm{N}}} \end{pmatrix} \tag{S1}$$

where [Mal$_{ref}$], [Pent$_{ref}$], [K_2CO_3], [P], [$U_{2,c}$], [U_2P_c], [UN], and [N] are the concentrations of substrates (Mal$_{ref}$ **3** and Pent$_{ref}$ **4**) and catalysts (K_2CO_3, Michael product, diUPy•K_2CO_3 complex, diUPy•product•K_2CO_3 complex, UPy-NaPy dimer, and free NaPy **1**), respectively, k_{K2CO3}, k_P, k_{U2}, k_{U2P}, k_{UN}, and k_N are their corresponding kinetic rate constants, and a_N and a_{UN} are the reaction orders of free NaPy and the UPy-NaPy dimer. Catalysts that have a subscripted *c* suffix have a separate complexation reaction with K_2CO_3, before they become catalytically active. We assume that the K_2CO_3 complex formation is not reversible, so only the forward reaction is included in the model.

The full set of ODEs describing the Michael addition of Mal$_{ref}$ **3** and Pent$_{ref}$ **4** was constructed with the assumption that the equilibria describing UPy-UPy and UPy-NaPy dimerization are not shifted during the reaction (Eq. S2). While ^{1}H NMR spectra obtained during the reaction show that the equilibria were shifting during the reaction, the magnitude of the shift could not be quantitatively determined due to deuteration. It was estimated that the shifts in equilibria had a maximum change of 30 %, and inclusion of the equilibria in the model did not lead to a significantly better fit. Therefore, they were omitted from the model.

$$\begin{aligned}
\frac{d}{dt}\left[\mathrm{substrate}\right] &= -2r_{M1} \\
\frac{d}{dt}\left[\mathrm{product}\right] &= r_{M1} - k_{U_2P_c}\left[\mathrm{U_2}\right]\left[\mathrm{P}\right]\left[\mathrm{K_2CO_3}\right] + k_{U_2P_c}\left[\mathrm{U_2P_c}\right]\left[\mathrm{substrate}\right]^2 \\
\frac{d}{dt}\left[\mathrm{U_2}\right] &= -k_{U_{2c}}\left[\mathrm{U_2}\right]\left[\mathrm{K_2CO_3}\right] + k_{U_2}\left[\mathrm{U_{2c}}\right]\left[\mathrm{substrate}\right]^2 \\
&\quad -k_{U_2P_c}\left[\mathrm{U_2}\right]\left[\mathrm{P}\right]\left[\mathrm{K_2CO_3}\right] + k_{U_2P_c}\left[\mathrm{U_2P_c}\right]\left[\mathrm{substrate}\right]^2 \\
\frac{d}{dt}\left[\mathrm{U_{2c}}\right] &= k_{U_{2c}}\left[\mathrm{U_2}\right]\left[\mathrm{K_2CO_3}\right] - k_{U_2}\left[\mathrm{U_{2c}}\right]\left[\mathrm{substrate}\right]^2 \\
\frac{d}{dt}\left[\mathrm{U_2P_c}\right] &= k_{U_2P_c}\left[\mathrm{U_2}\right]\left[\mathrm{P}\right]\left[\mathrm{K_2CO_3}\right] - k_{U_2P_c}\left[\mathrm{U_2P_c}\right]\left[\mathrm{substrate}\right]^2
\end{aligned} \tag{S2}$$

The ODEs were implemented in Matlab and solved using a stiff solver (*ode15s*). A Jacobian matrix was calculated using Matlab's symbolic math toolbox and provided to the solver to decrease the computational time.

A global analysis of all the data corresponding to the Michael addition of Mal_{ref} **3** and $Pent_{ref}$ **4** proved to be computationally expensive. Thus, three separate consecutive non-linear least square analyses were performed on orthogonal datasets (Figure S12A-C). The first dataset contains all catalysis experiments without any UPy or NaPy present, the second contains all experiments where UPy is present, and the third dataset contains all experiments with NaPy present. The optimized parameters obtained from the first dataset (k_{K2CO3} and k_P, Figure S12D) were used as fixed constants during the non-linear regression of the second dataset. Interestingly, no satisfactory fit of the third dataset could be achieved using the optimized parameters obtained from the second dataset (k_{U2}, k_{U2P}, k_{U2c}, and k_{U2Pc}) as fixed constants. Instead, those parameters were also set as free parameters in the non-linear least square analysis of the third dataset, in addition to the NaPy catalysis parameters (k_N, k_{UN}, and a_{UN}). The values of the optimized UPy parameters obtained from the regression of the third dataset were mostly higher compared to those obtained from the second dataset, which suggests that NaPy plays an activating role in UPy catalysis (Figure S12E-F).

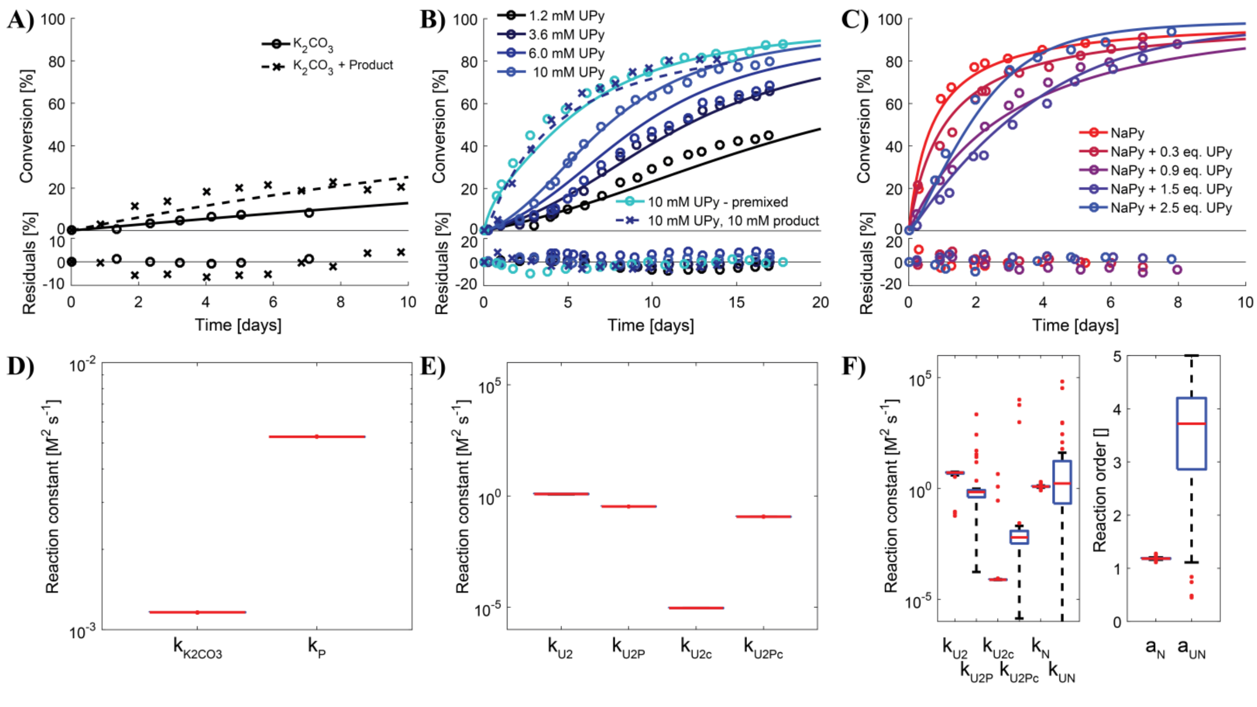

Figure S12: (A-C) The conversion of the Michael reaction between Mal_{ref} **3** and $Pent_{ref}$ **4** as obtained by ^{1}H NMR spectroscopy (markers), the best fit of the kinetic model (lines), and the corresponding residuals (markers) of datasets 1 (A; no UPy or NaPy present as catalysts), 2 (B; only UPy as catalyst) and 3 (C; NaPy present as catalyst). (D-F) Boxplots of the optimized parameters of all fits with a squared 2-norm residual within 5% of the best fit, corresponding to datasets 1 (D), 2 (E), and 3 (F). Note that the units of parameters K_N and K_{UN} are dependent on the exact values of the parameters a_N and a_{UN}, respectively.

*Influence of autocatalysis in the presence of NaPy **1***

Based on the first dataset, the autocatalytic activity of the product in the Michael addition of Mal_{ref} **3** and $Pent_{ref}$ **4** was found to be moderate compared to the activity of NaPy. To estimate the contribution of autocatalysis in the experiment where NaPy **1** is the primary catalyst, simulations were performed using the optimized parameters described above (Figure S13). In good agreement with the experimental results, NaPy catalysis is so efficient that it makes the contribution of autocatalysis negligible. The contribution by autocatalysis only exceeds 5 % at very low NaPy concentrations (< 1 mM; Figure S13C).

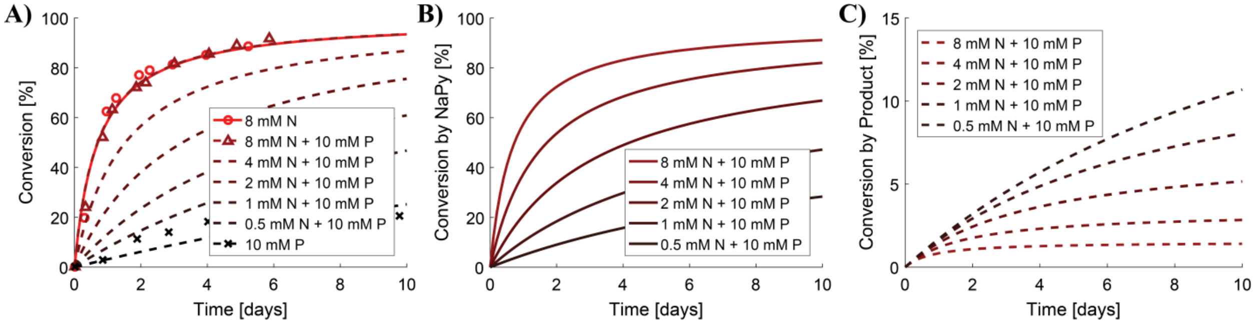

Figure S13: (A) experimental data of the conversion (markers) and simulated total conversion (lines) of the Michael reaction in the presence of varying amounts of NaPy **1**. (B-C) Predicted contributions of NaPy (B) and product (C) to the conversion of the Michael reaction. The activities of the product and NaPy are based on the optimized parameters from the first and third datasets, respectively. The concentrations of substrate (c = 4 mM) and K_2CO_3 (c = 36 mM) are the same in all simulations while the concentrations of NaPy (N) and product (P) are varied.

Validation for the inclusion of K_2CO_3 complexation

A fit of the second dataset without K_2CO_3 complexation was unable to describe the lag phase observed in the experimentally determined curves (Figure S14). An F-test was performed to compare the fits with and without K_2CO_3 complexation which revealed strong evidence for the inclusion of a separate complexation step for both the catalysts diUPy and diUPy•product ($p = 2.7 \times 10^{-34}$).

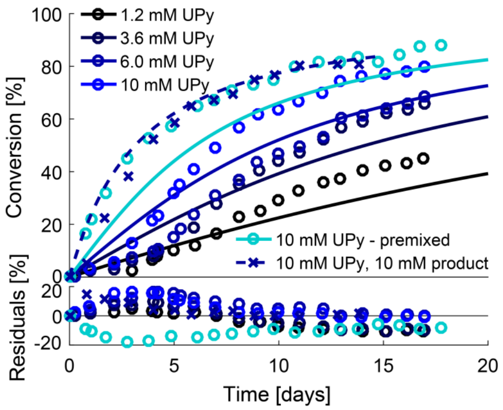

Figure S14: Fit of the second dataset without complexation reactions for the catalysts diUPy and diUPy•product.

Validation for the inclusion of the diUPy•product•K_2CO_3 complex

The inclusion of the diUPy•product•K_2CO_3 complex in the model was deemed necessary since a fit of the second dataset without the complex was unable to describe the data correctly. In particular, the experimental measurement in which pre-synthesized product was added at the beginning of the reaction consistently showed higher conversions compared to the optimized model prediction (Figure S15; crosses and dashed line). F-tests were used to compare the fits with and without diUPy autoinduction and confirmed the strong evidence for the inclusion of the complex ($p = 1.5 \times 10^{-42}$).

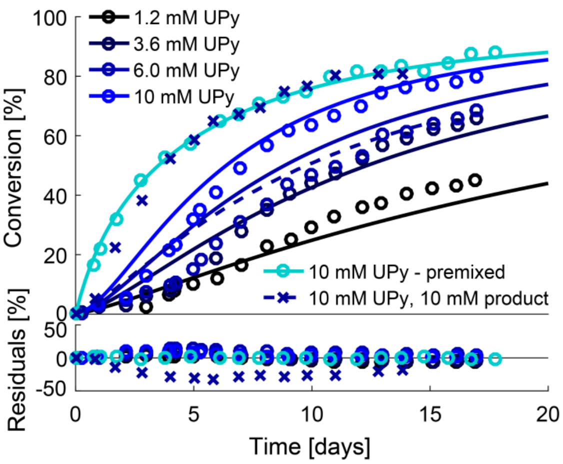

Figure S15: Fit of the second dataset without diUPy autoinduction.

*Kinetic model for the Michael addition of Mal_{ref} **3** and UPy_{pent} **5***

For the experiments where Mal_{ref} **3** and UPy_{pent} **5** are used as reagents, the overall reaction rate of the Michael addition was changed to include the change in catalysts as a function of conversion, *i.e.* the conversion of UPy_{pent} dimers to $UPy_{product}$ dimers (Eq. S3). Furthermore, in addition to the intermolecular catalysis by K_2CO_3 complexes, the possibility of intramolecular catalysis was added for diUPy•K_2CO_3 complexes comprising UPy_{pent} **5**.

$$r_{M2} = \left[\mathrm{Mal_{ref}}\right]\cdot\left[\mathrm{UD}\right]\cdot\begin{pmatrix} k_{\mathrm{K_2CO_3}}\left[\mathrm{K_2CO_3}\right] + k_{\mathrm{UD_2}}\left[\mathrm{UD_{2c}}\right] \\ +k_{\mathrm{UDUP}}\left[\mathrm{UDUP_c}\right] + k_{\mathrm{UP_2}}\left[\mathrm{UP_{2c}}\right] \\ +k_{\mathrm{UN}}\left[\mathrm{UN}\right]^{a_{\mathrm{UN}}} + k_{\mathrm{N}}\left[\mathrm{N}\right]^{a_{\mathrm{N}}} \end{pmatrix} + \left[\mathrm{Mal_{ref}}\right]\cdot\left(k_{\mathrm{UD_2,intra}}\left[\mathrm{UD_{2c}}\right] + k_{\mathrm{UDUP,intra}}\left[\mathrm{UDUP_c}\right]\right) \quad \text{(S3)}$$

where [UD], [$UD_{2,c}$], [$UDUP_c$], and [$UP_{2,c}$] are the concentrations of UPy_{pent}, diUPy_{pent}•K_2CO_3, UPy_{pent}•$UPy_{product}$•K_2CO_3, and di$UPy_{product}$•K_2CO_3, respectively, and k_{UD2}, k_{UDUP}, k_{UP2}, $k_{UD2,intra}$, and $k_{UDUP,intra}$ are the corresponding inter- and intramolecular rate constants.

The full set of ODEs describing the Michael addition of Mal_{ref} **3** and UPy_{pent} **5** was constructed with the assumption that the equilibria describing UPy-UPy and UPy-NaPy dimerization shift during the reaction as a consequence of the consumption of UPy_{pent} **5** and the production of $UPy_{product}$ **6** (Eq. S4, Figure S16A). To accommodate the inclusion of dimerization and its possibility to shift during the reaction, a simple mass balance for UPy_{pent} and $UPy_{product}$ dimerization was solved each time the numerical values of the ODEs were calculated. Solving the mass balance yields [UD_2], [UDUP], and [UP_2] during the course of the reaction, which are used to calculate the reaction rates for the formation of [$UD_{2,c}$], [$UDUP_c$], and [$UP_{2,c}$]. A good description of the experimental data could only be obtained when we assumed that K_2CO_3 complexation influences UPy-UPy and UPy-NaPy equilibria. The ODEs were implemented in Matlab and solved using a stiff solver (*ode15s*).

$$
\begin{aligned}
r_{\mathrm{UD_2,c}} &= k_{UD_2c}\left[\mathrm{UD_2}\right]\left[\mathrm{K_2CO_3}\right] \\
r_{\mathrm{UDUP,c}} &= k_{\mathrm{UDUPc}}\left[\mathrm{UDUP}\right]\left[\mathrm{K_2CO_3}\right] \\
r_{\mathrm{UP_2,c}} &= k_{\mathrm{UP_2c}}\left[\mathrm{UP_2}\right]\left[\mathrm{K_2CO_3}\right] \\
r_{\mathrm{UD_2,inter}} &= k_{UD_2}\left[\mathrm{UD_{2c}}\right]\left[\mathrm{Mal_{ref}}\right]\left[\mathrm{UD}\right] \\
r_{\mathrm{UDUP,inter}} &= k_{\mathrm{UDUP}}\left[\mathrm{UDUP_c}\right]\left[\mathrm{Mal_{ref}}\right]\left[\mathrm{UD}\right] \\
r_{\mathrm{UP_2,inter}} &= k_{\mathrm{UP_2}}\left[\mathrm{UP_{2c}}\right]\left[\mathrm{Mal_{ref}}\right]\left[\mathrm{UD}\right] \\
r_{\mathrm{UD_2,intra}} &= k_{\mathrm{UD_2,intra}}\left[\mathrm{UD_{2c}}\right]\left[\mathrm{Mal_{ref}}\right] \\
r_{\mathrm{UDUP,intra}} &= k_{\mathrm{UDUP,intra}}\left[\mathrm{UDUP_c}\right]\left[\mathrm{Mal_{ref}}\right] \\
\frac{d}{dt}\left[\mathrm{Mal_{ref}}\right] &= -r_{M2} \\
\frac{d}{dt}\left[\mathrm{UD}\right] &= -r_{M2} - 2r_{\mathrm{UD_2,c}} + 2r_{\mathrm{UD_2,inter}} \\
&\quad - r_{\mathrm{UDUP,c}} + r_{\mathrm{UDUP,inter}} \\
&\quad + r_{\mathrm{UD_2,intra}} \\
\frac{d}{dt}\left[\mathrm{UP}\right] &= +r_{M2} - r_{\mathrm{UDUP,c}} + r_{\mathrm{UDUP,inter}} \\
&\quad - 2r_{\mathrm{UP_2,c}} + 2r_{\mathrm{UP_2,inter}} \\
&\quad + 2r_{\mathrm{UDUP,intra}} \\
\frac{d}{dt}\left[\mathrm{UD_{2c}}\right] &= +r_{\mathrm{UD_2,c}} - r_{\mathrm{UD_2,inter}} - r_{\mathrm{UD_2,intra}} \\
\frac{d}{dt}\left[\mathrm{UDUP_c}\right] &= +r_{\mathrm{UDUP,c}} - r_{\mathrm{UDUP,inter}} - r_{\mathrm{UDUP,intra}} \\
\frac{d}{dt}\left[\mathrm{UP_2P_c}\right] &= r_{\mathrm{UP_2,c}} - r_{\mathrm{UP_2,inter}}
\end{aligned}
\tag{S4}
$$

To describe the behavior of UPy_{pent} **5**, a non-linear least square optimization was performed using the data from the experiments where the concentrations of Mal_{ref} **3** and UPy_{pent} **5** are changed simultaneously, using k_{UD2}, k_{UDUP}, k_{UP2}, $k_{UD2,intra}$, $k_{UDUP,intra}$, k_{UD2c}, k_{UDUPc}, and k_{UP2c} as free parameters (Figure 5B and S16B-C). While the optimized model can accurately describe both the initial lag phase and the concentration independence, some deviations between the fit and the data are observed at high conversions, which are attributed to small variations in experimental conditions. In line with the strong rate acceleration during the course of the reaction, the values of the optimized parameters increase going from the kinetic constants of UPy_{pent} dimers (UD_2) to those of the UPy_{pent}-$UPy_{product}$ (UDUP), to those of $UPy_{product}$ dimers (UP_2). Thus, while UPy_{pent} is converted to $UPy_{product}$, the simulated reaction rate increases due to the increase in the values of the optimized kinetic constants. Model predictions on the influence of NaPy on the Michael addition between Mal_{ref} **3** and UPy_{pent} **5** were performed using the optimized parameters of the NaPy catalysis in the Michael addition of Mal_{ref} **3** and $Pent_{ref}$ **4** (Figure S12F). Equation S3 was adjusted with the appropriate terms and the mass balances were adjusted to include UPy-NaPy heterodimerization.

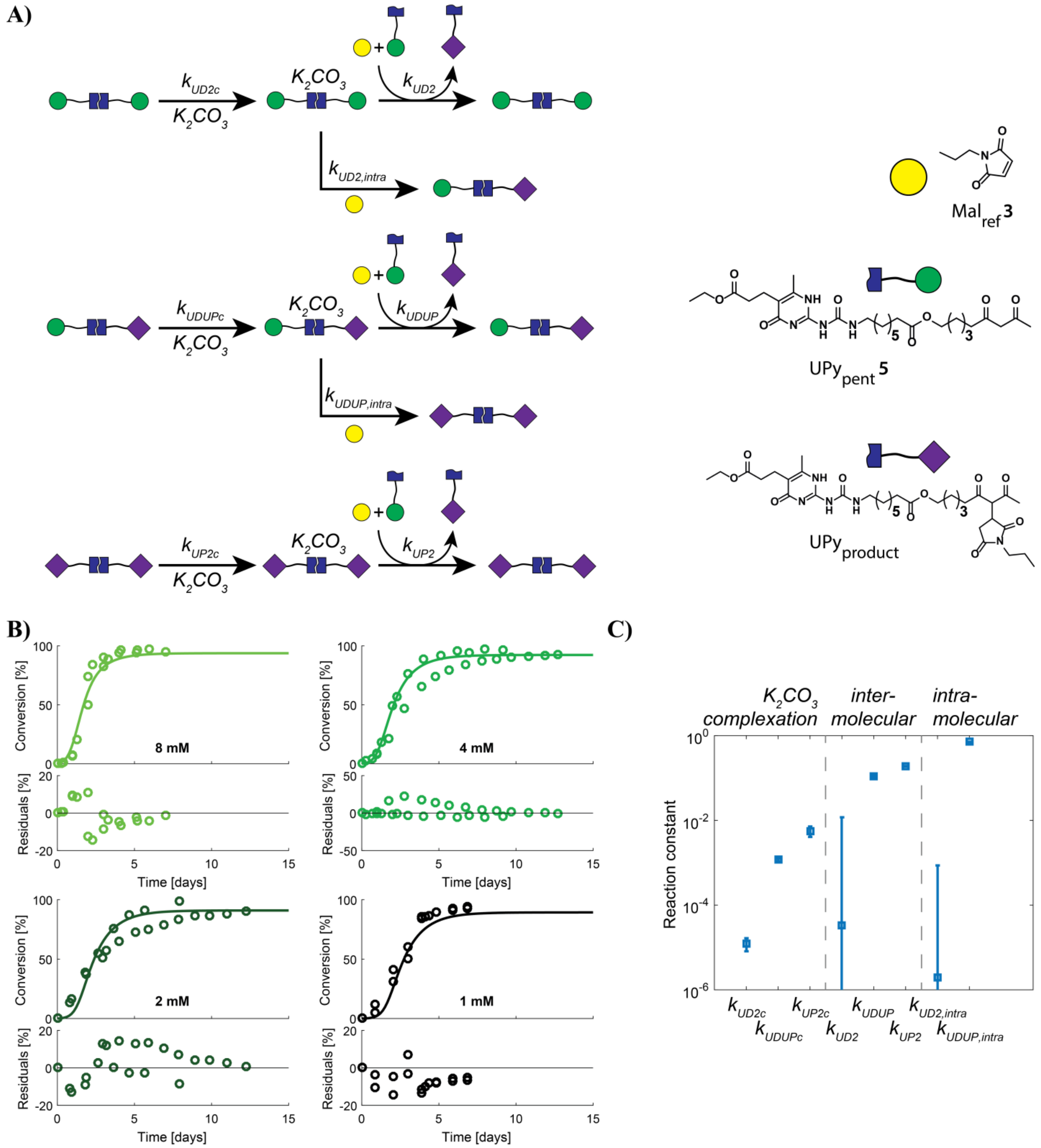

Figure S16: (A) Schematic of the reactions included in the model for the Michael addition between Mal$_{ref}$ **3** and UPy$_{pent}$ **5** without NaPy catalysis. (B) The conversion of the Michael reaction between Mal$_{ref}$ **3** and UPy$_{pent}$ **5** as obtained by ^{1}H NMR spectroscopy (markers; measured in duplo), the best fit of the kinetic model (lines), and the corresponding residuals (markers) at various total concentrations. (C) Optimized parameter values and 95% confidence interval of the best fit.
